# The phosphatidylcholine transfer protein StarD7 is important for myogenic differentiation in mouse myoblast C2C12 cells and human primary skeletal myoblasts

**DOI:** 10.1038/s41598-020-59444-y

**Published:** 2020-02-18

**Authors:** Yasuhiro Horibata, Satomi Mitsuhashi, Hiroaki Shimizu, Sho Maejima, Hirotaka Sakamoto, Chieko Aoyama, Hiromi Ando, Hiroyuki Sugimoto

**Affiliations:** 1 Department of Biochemistry, Dokkyo Medical University School of Medicine, 880 Kitakobayashi, Mibu Tochigi, 321-0293 Japan; 2 Department of Human Genetics, Yokohama City University Graduate School of Medicine, Fukuura 3-9, Kanazawa-ku Yokohama, 236-0004 Japan; 3 Ushimado Marine Institute (UMI), Graduate School of Natural Science and Technology, Okayama University, Ushimado, Setouchi Okayama, 701-4303 Japan

**Keywords:** Phospholipids, Myosin

## Abstract

StarD7 is a phosphatidylcholine (PC)-specific lipid transfer protein essential for the maintenance of mitochondrial PC composition, morphogenesis, and respiration. Here, we studied the role of StarD7 in skeletal myoblast differentiation using mouse myoblast C2C12 cells and human primary myoblasts. Immunofluorescence and immuno-electron microscopy revealed that StarD7 was distributed in the cytosol, inner mitochondria space, and outer leaflet of the outer mitochondrial membrane in C2C12 cells. Unlike human kidney embryonic cell line HEK293 cells, the mitochondrial proteinase PARL was not involved in the processing and maturation of StarD7 in C2C12 cells. StarD7 was constantly expressed during myogenic differentiation of C2C12 cells. The siRNA-mediated knockdown of StarD7 in C2C12 cells and human primary myoblasts significantly impaired myogenic differentiation and reduced the expression of myomaker, myomerger and PGC-1α. The reduction in mitochondrial PC levels and oxygen consumption rates, decreased expression of myomaker, myomerger and PGC-1α, as well as impaired myogenic differentiation, were completely restored when the protein was reintroduced into *StarD7*-knockout C2C12 cells. These results suggest that StarD7 is important for skeletal myogenesis in mammals.

## Introduction

Mitochondrial membranes are composed of two phospholipid bilayers, the inner mitochondrial membrane (IMM) and the outer mitochondrial membrane (OMM). These mitochondrial membranes are essential for compartmentalization of the organelle and for various mitochondrial functions. More than half of the phospholipids in mitochondrial membranes are phosphatidylcholine (PC), followed by phosphatidylethanolamine (PE) (30–40%), cardiolipin (CL) (5–15%), phosphatidylinositol (PI) (2–9%), phosphatidylserine (PS) (1%) and phosphatidylglycerol (PG) (<1%). Mitochondria have enzymes for the biosynthesis of PE, CL and PG but lack the enzymes required for PC, PI and PS synthesis. These lipids are therefore likely supplied from organelles including as the endoplasmic reticulum (ER) which contain the biosynthesis systems for these phospholipids^1,2^.

Several previous studies have proposed that phospholipids are transferred between the ER and mitochondria *via* ER-mitochondria contact sites. In yeast, the formation of contact sites is mediated by a protein complex, the ER-mitochondria encounter structure (ERMES)^3^. ERMES comprises four core subunits: Mmm1, Mdm10, Mdm34 and Mdm12. Recent studies demonstrated that the Mmm1-Mdm12 complex directly facilitates PS/PE transfer between the ER and mitochondria *in vitro* and *in vivo*^4–6^. In mammals, ER-mitochondria contact sites are referred to as ER mitochondria-associated membranes (MAM). Several proteins such as mitofusin 2 (MFN2)^7,8^, glucose-regulated protein 75 (GRP75)^9^, mitochondrial fission 1 protein (Fis1) and B-cell receptor-associated protein 31 (Bap31)^10^ have been reported to be implicated in the formation of membrane contact sites. A recent study showed that MFN2 directly and specifically binds to PS, and mediates PS transfer between the ER and mitochondria^11^. However, the molecular mechanism by which PC and PI transfer at these sites remains unknown.

Steroidogenic acute regulatory protein-related lipid transfer (START) domains are lipid binding/transfer domains involved in the intracellular lipid transport of phospholipids, sterols and sphingolipids. Fifteen mammalian proteins with this domain have been identified to date and are named StarD1-StarD15^12,13^. We previously reported that StarD7 is located in both the cytoplasm and mitochondria, specifically binds/transfers PC *in vitro*, and facilitates the transfer of extracellularly added PC to mitochondria in mouse HEPA-1 cells (a cell line derived from hepatocellular carcinoma)^14^. In mitochondria from HEPA-1 cells, the protein is localized both in the inner mitochondria and on the OMM. We found that a part of StarD7 on the OMM is co-localized with MFN2, and proposed that the protein might mediate PC transfer between the ER and mitochondria at the membrane contact sites^15^ and that the loss of StarD7 results in reduced mitochondrial PC content^16^. Recently, another group demonstrated that StarD7 is proteolytically processed to its mature form by presenilin-associated rhomboid-like protein (PARL), a rhomboid family protease which distributes in the IMM in HeLa and HEK293 human cell lines^17^. They reported that StarD7 is distributed in the mitochondrial intermembrane space (IMS), and proposed that the protein shuttles PC between the OMM and IMM. Interestingly, a deficiency of StarD7 resulted in incomplete formation of cristae and impairment of mitochondrial respiration, suggesting that the protein is required not only for maintaining the level of PC in mitochondria but also for mitochondrial morphogenesis and function^16–18^.

Several studies have reported the biological function and significance of StarD7 in cell lines and animal models. In HepG2 cells, silencing of StarD7 caused ER stress and the production of reactive oxygen species (ROS)^19^. In trophoblast JEG-3 cells, the expression of StarD7 was modulated by glucose by Wnt/β-catenin signaling *via* activation of the hexosamine biosynthesis pathway^20^. StarD7 knockdown in the cells reduced the expression of multidrug transporter protein, cell migration, and cell proliferation^21^. Hetero *StarD7*-knockout (KO) mice showed enhanced allergic response in lung and skin^22^. Studies on a bronchiolar epithelial cell line (BEAS-2B) and lung epithelial cell-specific conditional KO mice revealed that mitochondrial dysfunction due to the lack of StarD7 caused defects in the epithelial barrier^18^. However, the importance of a biological role for StarD7 in skeletal muscle remains poorly understood.

In this study, we determined the contribution of StarD7 to myogenic differentiation in skeletal myoblasts. The absence of StarD7 in siRNA-mediated knockdown (KD) mouse myoblast C2C12 cells and human primary myoblasts caused significant impairment of myotube formation following differentiation stimulation. The expression of myomaker and myomerger, and peroxisome proliferator activated receptor gamma coactivator 1 alpha (PGC-1α), those are essential for myoblast fusion and mitochondrial biogenesis, respectively, were significantly down-regulated in these knockdown cells. Defects in myotube formation, reduced levels of mitochondrial PC, decreased respiration, and the expression of myomaker, myomerger and PGC-1α were completely recovered when StarD7 was reintroduced into *StarD7*-KO C2C12 cells. These results demonstrate for the first time that StarD7 is important for myogenic differentiation in mammalian myoblasts.

## Results

### Subcellular and sub-mitochondrial distribution of StarD7 in C2C12 cells

**Figure 1:**
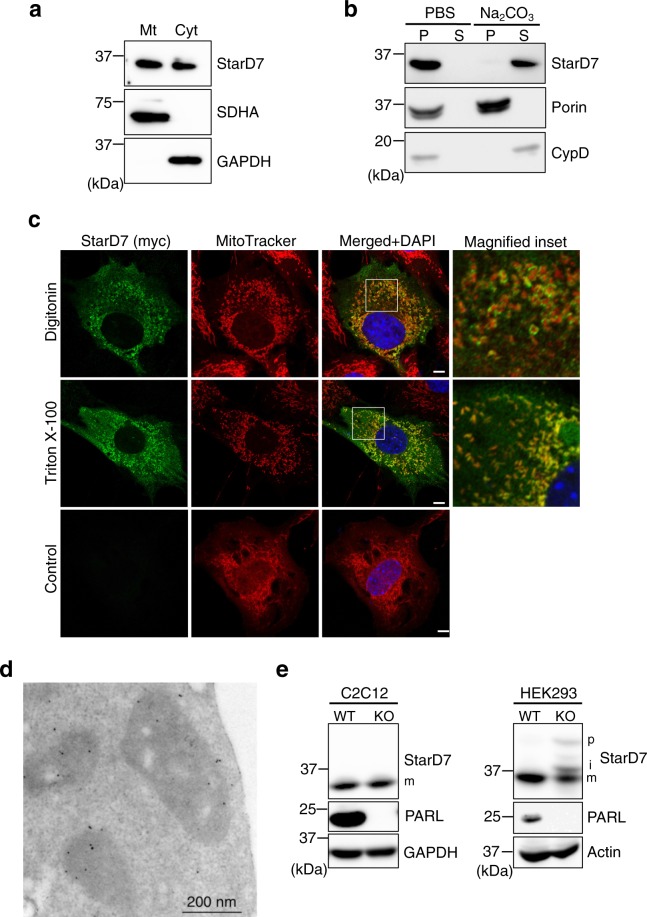
Sub-mitochondrial distribution and PARL-independent maturation of StarD7 in C2C12 cells. (**a**) Mitochondria and cytosol were separated from C2C12 cells by subcellular fractionation and analyzed by western blotting using anti-StarD7, -SDHA and -GAPDH antibodies. Mt and Cyt indicate mitochondria and cytosol, respectively. (**b**) Mitochondria isolated from the cells were treated with PBS or alkaline buffer (Na_2_CO_3_), then the pellet (P) and supernatant (S) were separated by centrifugation. These fractions were analyzed by western blotting with anti-StarD7, porin and CypD antibodies. Porin is a membrane-integrated protein and CypD is a matrix protein. (**c**) C2C12 cells in proliferation condition were transfected with the expression vector for StarD7 fused with a myc tag at the C-terminus. Cells were permeabilized with 0.005% digitonin (w/v) or 0.1% Triton X-100 (w/v), then immunostained with anti-myc antibody (green). Control means cells without detergent treatment. Mitochondria and nuclei were stained with MitoTracker Red (red) and DAPI (blue), respectively. *Bars* indicate 5 μm. (**d**) C2C12 cells were transfected with the expression vector for StarD7 fused with a V5 tag at the C-terminus. After fixation, sections were stained with anti-V5 antibody followed by secondary gold-conjugated antibody. (**e**) Cell lysates from WT and *PARL*-KO C2C12 and HEK 293 cells were separated by SDS-PAGE, then the proteins were analyzed by western blotting using anti-StarD7 and PARL antibodies. p, precursor; i, intermediate; m, mature form of StarD7. GAPDH and actin were used as protein loading controls.

Previous studies showed that StarD7 is distributed in both the cytosol and mitochondria in several cell lines, including HEPA-1, HeLa, and HEK293 cells^14,15,17^. Consistent with these results, a subcellular fractionation experiment revealed that StarD7 is distributed in both the cytosolic and mitochondrial fractions from C2C12 cells (Fig. 1a). Our previous alkaline carbonate extraction assay study showed that StarD7 is a mitochondrial membrane-integrated protein in HEPA-1 cells^15^ and thus here we analyzed the membrane integration of StarD7 in C2C12 cells. After purification, the mitochondria were treated with alkaline carbonate solution (pH 11.5) to release the matrix proteins and membrane-associated proteins from the mitochondrial membrane. As shown in Fig. 1b, the membrane-integrated protein porin was detected in the pellet (P) whereas cyclophilin D (CypD), a mitochondrial matrix protein, and StarD7 were recovered in the supernatant (S). These results suggest that unlike HEPA-1 cells, StarD7 is not integrated into mitochondrial membranes in C2C12 cells.

Next, we analyzed the sub-mitochondrial distribution of StarD7 using immunofluorescence with C2C12 cells. After transfection with an expression vector containing StarD7 fused with a myc-tag at the C-terminus, the cells were fixed with paraformaldehyde. Digitonin has high affinity for cholesterol and preferentially extract cholesterol from plasma membrane, in which cholesterol is enriched relative to intracellular compartments^23–27^. C2C12 cells were treated with 0.005% digitonin (w/v) or 0.1% Triton X-100 (w/v) to permeabilize the plasma membrane or cell membrane, respectively. The integrity of the mitochondrial membrane was verified by immunostaining with translocator of the outer membrane 70 (TOM70), YME1 like 1 ATPase (YME1L1), and succinate dehydrogenase complex iron sulfur subunit A (SDHA). These protein epitopes localized on the cytoplasmic face of the OMM, in the IMS, and inside the matrix, respectively. The sub-mitochondrial topologies of these proteins and the epitope positions are summarized in Fig. S1a,b. Using C2C12 cells, TOM70, but not YMEL1 and SDHA, was visualized after digitonin treatment, showing that only the plasma membrane was permeabilized by digitonin treatment and that the OMM was not permeabilized (Fig. S1c). In contrast, Triton X-100 treatment resulted in visualization of all the proteins, indicating that both the mitochondrial and plasma membranes were permeabilized. As shown in Fig. 1c, StarD7 was visualized in both the cytosol and peripheral outer membrane of the mitochondria after digitonin permeabilization in C2C12 cells, suggesting that the C-terminal START domain of StarD7 is localized on the cytoplasmic face of the OMM. StarD7 distributed in the inner mitochondrial area was visualized in Triton X-100-permeabilized cells. Similar results were obtained using HEPA-1 cells (Fig. S2).

We also analyzed the sub-mitochondrial distribution of StarD7 using immuno-electron microscopy. After transfection with an expression vector containing StarD7 fused with a V5-tag at the C-terminus, the cells were fixed with paraformaldehyde-glutaraldehyde solution. The sections were incubated with a primary anti-V5 antibody, followed by a secondary gold-conjugated antibody. As shown in Fig. 1d (C2C12 cells) and Fig. S3 (HEPA-1 cells), gold particles were observed in both the inner mitochondrial area and on the OMM. These results support our finding that StarD7 is localized on both the outer leaflet of the OMM and in the inner mitochondria in C2C12 and HEPA-1 cells.

### PARL is not involved in the maturation of StarD7 in C2C12 and HEPA-1 cells

Recently, PARL was identified as the IMM protease responsible for the processing and maturation of StarD7^17^ in human cell lines such as HeLa and HEK293, and that PARL-mediated cleavage regulates protein localization between the mitochondria and the cytoplasm. We prepared *PARL*-deficient C2C12 and HEK293 cells using the CRISPR-Cas9 system to assess the cleavage of StarD7. Consistent with the previous report by Saita *et al*.^17^, precursor (p) and intermediate-sized forms (i) of StarD7 partially accumulated in *PARL*-KO HEK293 cells, indicating that part of the protein is processed by PARL in these cells (Fig. 1e, right panel). In contrast, no accumulation of the p and i forms of StarD7 was detected in PARL-deficient C2C12 cells (Fig. 1e, left panel) or in PARL-deficient HEPA-1 cells (Fig. S4a).

Saita and coworkers also reported that the major portion of StarD7 accumulated in the mitochondria of PARL-deficient HeLa cells^17^. We therefore analyzed the subcellular distribution of StarD7 in *PARL*-KO C2C12 cells using immunofluorescence (Fig. S4b). We also calculated the percentage of cells showing StarD7 localization only in mitochondria or in both mitochondria and cytosol (Fig. S4c). These results showed that a lack of PARL had no significant effect on the subcellular distribution of StarD7 in C2C12 cells, suggesting that PARL is not involved in the maturation of StarD7, at least in cells of the two mouse cell lines C2C12 and HEPA-1.

### StarD7 is constantly expressed during myogenic differentiation in C2C12 cells

**Figure 2:**
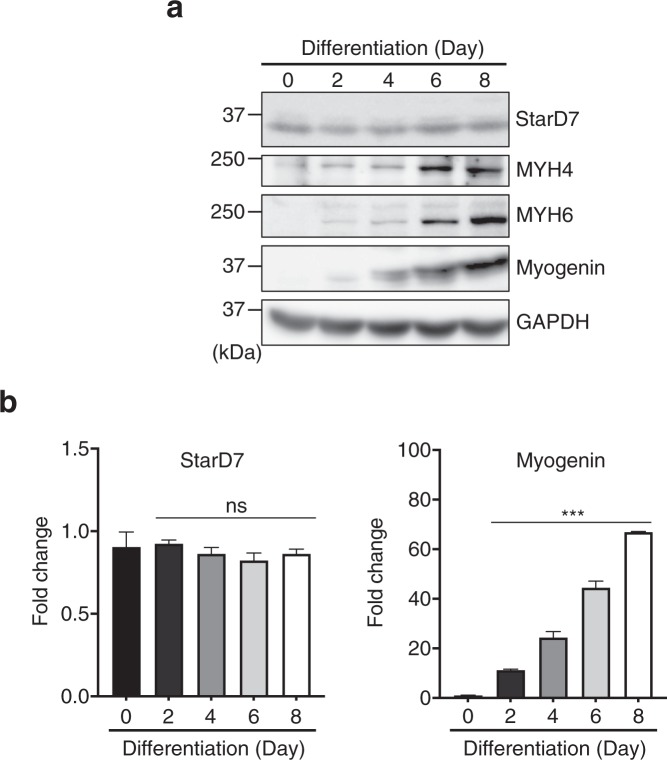
Expression of StarD7 during myogenic differentiation in C2C12 cells. (**a**) The protein levels of StarD7, MYH4, MYH6, and myogenin in C2C12 cells cultured in differentiation medium for the days indicated were analyzed by western blotting. GAPDH was used as a protein loading control. (**b**) The mRNA levels of StarD7 and myogenin in C2C12 cells differentiated for the days indicated were quantified by qPCR. Values shown are means ± S.D. ***P < 0.001 as compared with day 0. ns indicates not significant as compared with day 0 (one-way ANOVA with Tukey’s post hoc test).

Culturing C2C12 cells in differentiation medium with a low serum concentration accelerates the expression of myogenic regulator factors (MRF) such as MyoD and myogenin, which induce the expression of myosin heavy chain (MYH), resulting in the cells differentiating into multinucleated myotubes. We investigated whether the expression of StarD7 is regulated by these MRFs by analyzing the level of StarD7 during myogenin differentiation. As shown in Fig. 2a, the protein levels of MYH4, MYH6, and myogenin increased during differentiation over a 8 day period whereas the level of StarD7 protein remained constant during differentiation. We also analyzed mRNA levels using qPCR. As shown in Fig. 2b, the expression of StarD7 was stable whereas the mRNA levels of myogenin was up-regulated during differentiation.

### The loss of StarD7 impairs myoblast differentiation in C2C12 cells

**Figure 3:**
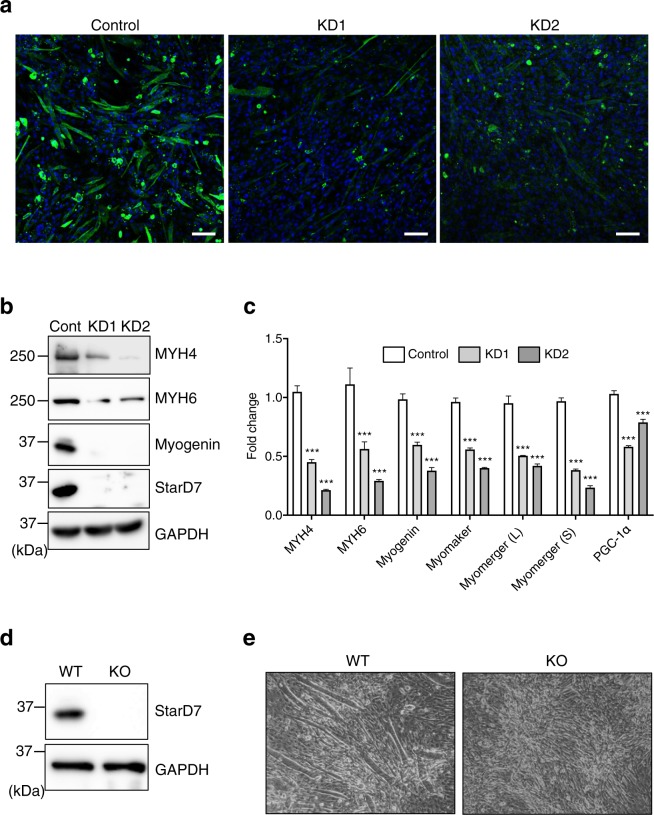
The loss of StarD7 impaired myogenic differentiation in C2C12 cells. (**a**) After transfection with siRNAs against StarD7 (KD1 or KD2) or control siRNA, C2C12 cells were cultured in differentiation medium for 5 days, then immunostained with anti-MYH6 antibody (green). Nuclei were stained with DAPI (blue). *Bars* indicate 50 μm. (**b**) Protein levels of MYH4, MYH6, myogenin and StarD7 were analyzed by western blotting. GAPDH was used as a protein loading control. (**c**) The mRNA levels of MYH4, MYH6, myogenin, myomaker, myomerger (L), myomerger (S) and PGC-1α were quantified by qPCR. Data were normalized to the GAPDH level. Values shown are means ± S.D. from three independent culture dishes. ***P < 0.001 as compared with control siRNA (one-way ANOVA with Tukey’s post hoc test). (**d**) Protein levels of StarD7 in WT and *StarD7*-KO C2C12 cells were analyzed by western blotting. (**e**) WT or *StarD7*-KO C2C12 cells were cultured in differentiation medium for 5 days and phase contrast images were obtained.

We clarified the importance of StarD7 for myogenic differentiation in C2C12 cells by preparing StarD7 knocked down cells (KD) by siRNA. After transfection with control or StarD7-targeted siRNAs (KD1 and KD2), the cells were cultured in differentiation medium for 5 days and the expression of MYH6 was analyzed by immunofluorescence. As shown in Fig. 3a, the number of MYH6-positive KD1 and KD2 cells clearly decreased compared to control cells after differentiation. The protein levels of MYH4, MYH6, myogenin, and StarD7 after differentiation were examined by western blotting. As shown in Fig. 3b, StarD7 was efficiently silenced by siRNA compared with control cells. As expected, the levels of MYH4 and MYH6 were clearly decreased in the KD cells, and interestingly, the induction of myogenin was strongly inhibited. Next, we performed qPCR to assess the mRNA levels of these differentiation markers. As shown in Fig. 3c, the mRNA levels of MYH4, MYH6, and myogenin were significantly decreased in StarD7- KD C2C12 cells. We also quantified the mRNA levels of myomaker and both the short (S) and long (L) transcripts of myomerger (also referred to as minion or myomixer) during differentiation. These are membrane proteins required for plasma membrane remodeling and myoblast fusion. We monitored the level of PGC-1α, a master regulator for mitochondrial biogenesis as well. As shown in Fig. 3c, the expression of these genes was significantly reduced in the KD cells compared to control cells.

We analyzed the role of StarD7 in myoblast differentiation more precisely by preparing *StarD7*-KO C2C12 cells using CRISPR/Cas9 genome editing. As shown in Fig. 3d, no StarD7 protein band was detected in the KO cells. Next, we cultured WT and KO cells in differentiation medium for 5 days and assessed myotube formation. As shown in Fig. 3e, no myotubes were observed in the KO cells. These findings suggest that StarD7 in required for myogenic differentiation in C2C12 cells.

### Impaired myogenic differentiation in StarD7-KO C2C12 cells is recovered by the reintroduction of StarD7

**Figure 4:**
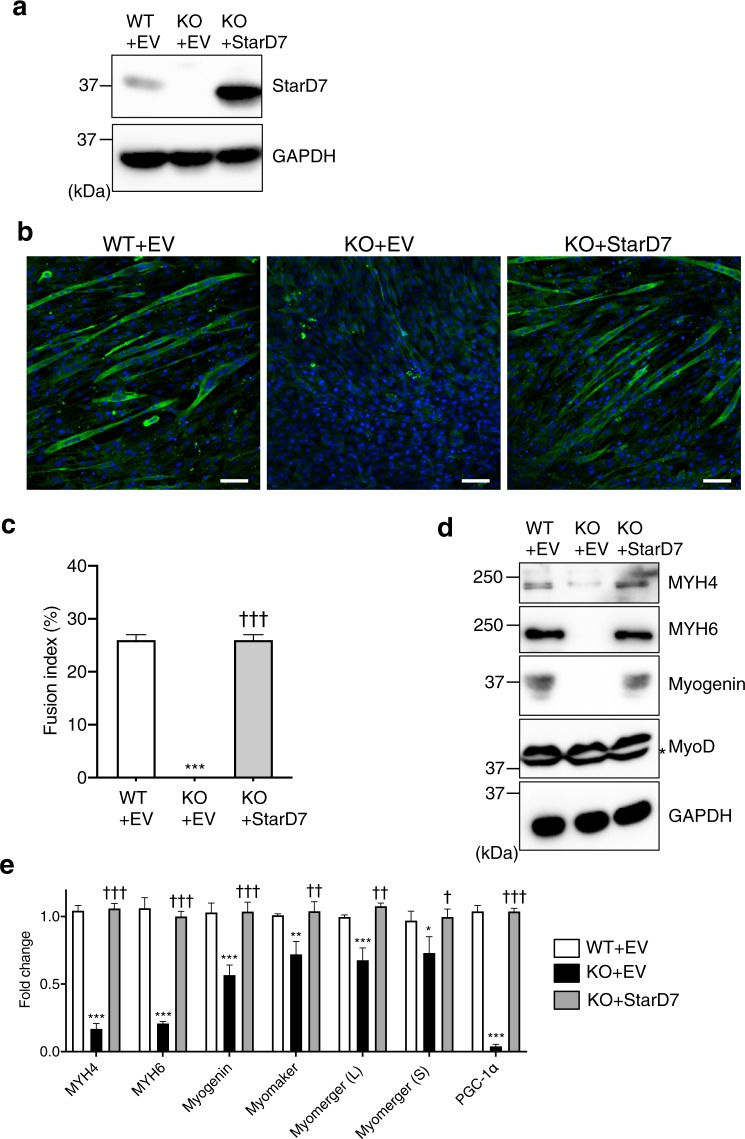
Reintroduction of StarD7 into the *StarD7*-KO C2C12 cells restored myogenic differentiation. (**a**) Cell lysates from WT + EV (empty vector), KO + EV, and KO + StarD7 C2C12 cells were separated by SDS-PAGE and analyzed by western blotting using anti-StarD7 antibody. GAPDH was used as a protein loading control. (**b**) WT + EV, KO + EV, and KO + StarD7 C2C12 cells were cultured in differentiation medium for 5 days, then immunostained with anti-MYH6 antibody (green). Nuclei were stained with DAPI (blue). *Bars* indicate 50 μm. (**c**) The fusion indexes of C2C12 cells were calculated, and are presented as the means ± S.D. ***P < 0.001 as compared with WT + EV cells, and ^†††^*P* < 0.001 as compared with KO + EV cells (one-way ANOVA with Tukey’s post hoc test). (**d**) After inducing differentiation, the amounts of MYH4, MYH6, myogenin and myoD protein were analyzed by western blotting. Asterisk indicates a non-specific band. (**e**) The mRNA levels of MYH4, MYH6, myogenin, myomaker, myomerger (L), myomerger (S) and PGC-1α were quantified by qPCR after inducing differentiation. Data were normalized to the GAPDH level. Values shown are means ± S.D. from three independent culture dishes. *P < 0.05, **P < 0.01, and ***P < 0.001 as compared with WT + EV cells, and ^†^*P* < 0.05, ^††^*P* < 0.01, and ^†††^*P* < 0.001 as compared with KO + EV cells (one-way ANOVA with Tukey’s post hoc test).

To exclude the possibility of off-target effects, we reintroduced an expression vector for StarD7 into the KO cells to establish *StarD7*-KO cells stably expressing StarD7 (KO + StarD7). At the same time, we also established WT and KO cells expressing an empty vector (EV) (WT + EV and KO + EV, respectively). The protein expression of StarD7 in the three cell types was checked by western blotting. As shown in Fig. 4a, KO + StarD7 cells showed restored protein expression of StarD7. These cells were then cultured in differentiation medium for 5 days to induce myogenic differentiation and the protein level of MYH6 was assessed by immunofluorescence. As shown in Fig. 4b, the number MYH6-positive KO + EV cells was greatly reduced in comparison to WT + EV cells. It should be noted that the expression of MYH6 was recovered in KO + StarD7 cells. We calculated the fusion index (the percentage of the number of nuclei in MYH6-positive cells with 3 or more nuclei to the total number of nuclei). As shown in Fig. 4c, the fusion index in WT + EV, KO + EV, and KO + StarD7 was 25.7%, 0%, and 25.8%, respectively. Furthermore, Fig. 4d shows that the expression of MYH4, MYH6, and myogenin was strongly decreased in KO + EV cells but expression was clearly recovered in KO + StarD7 cells. MyoD expression was not affected by the loss of StarD7. Next, we performed qPCR to assess the mRNA levels of these differentiation markers. As shown in Fig. 4e, the mRNA levels of MYH4, MYH6, and myogenin were significantly decreased in KO + EV cells, and were restored in KO + StarD7 cells. The decrease in the mRNA levels of myomaker, myomerger, and PGC-1α in KO + EV cells were also restored in KO + StarD7 cells. We also monitored the myogenic differentiation of another KO + StarD7 clone, #2 (Fig. S5). For reasons currently unclear, this clone showed higher myogenin induction (Fig. S5a) and shorter myotubes (Fig. S5c,d) than WT-EV. These results strongly support our findings that StarD7 is important for myogenic differentiation in C2C12 cells.

### StarD7 is important for maintaining mitochondrial PC levels and respiration in C2C12 cells

**Figure 5:**
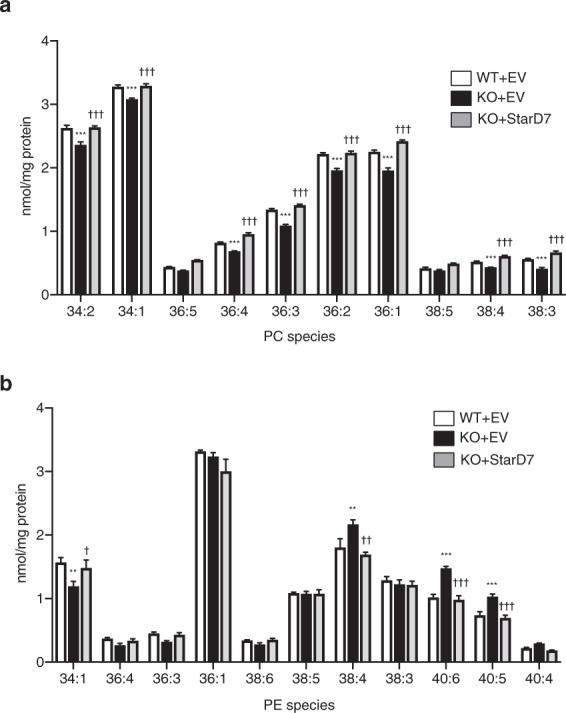
Reintroduction of StarD7 into the *StarD7*-KO C2C12 cells restored mitochondrial PC composition. Mitochondria were purified from WT + EV, KO + EV, and KO + StarD7 C2C12 cells, phospholipids were extracted, and the amounts of (**a**) PC and (**b**) PE were determined using LC-MS/MS with multiple reaction monitoring. Values were normalized against the amount of mitochondrial protein. The results shown are from one representative experiment of five experiments giving similar results. Data are means ± S.D. from quadruplet analyses of one experiment. **P < 0.01 and ***P < 0.001 as compared with WT + EV cells. ^†^*P* < 0.05, ^††^*P* < 0.01, and ^†††^*P* < 0.001 as compared with KO + EV cells (one-way ANOVA with Tukey’s post hoc test).

It was previously demonstrated that StarD7 is required for maintaining the content of mitochondrial PC in cell lines such as HEPA-1^16^ and HEK293^17^. To confirm the importance of StarD7 for maintaining mitochondrial PC levels in C2C12 cells as well, mitochondria were obtained from WT + EV, KO + EV, and KO + StarD7 cells using a Percoll/Nycodenz discontinuous density gradient. After the phospholipids were extracted, the concentrations of PC and PE were quantified by liquid chromatography-tandem mass spectrometry (LC-MS/MS). As shown in Fig. 5a, the proportions of the various PC species were significantly reduced in KO + EV cells compared with WT + EV cells. The reduced PC level was restored in KO + StarD7 cells. In our previous study, we found that the level of PE (18:0–20:4) was increased in StarD7-deficient HEPA-1 cells as compared with WT cells^16^. Similar to these previous results, we found an increase in mitochondrial PE (38:4, 40:6 and 40:5) in the KO + EV cells as compared with WT + EV (Fig. 5b).

**Figure 6:**
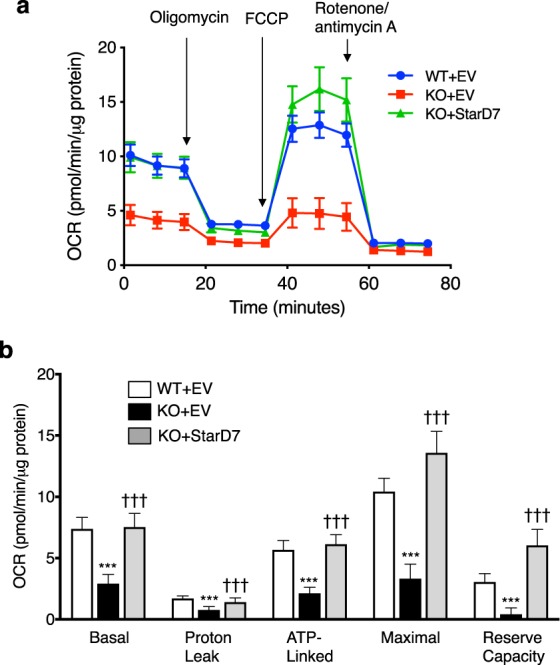
Reintroduction of StarD7 into *StarD7*-KO C2C12 cells restored mitochondrial respiration. The real-time oxygen consumption rate (OCR) of WT + EV, KO + EV, and KO + StarD7 C2C12 cells in proliferation condition was determined using a Seahorse XFp analyzer. (**a**) Raw data of OCR, (**b**) basal respiration, proton leak, ATP-linked respiration (oligomycin-sensitive OCR), maximal mitochondrial respiration (FCCP–stimulated OCR), and reserve capacity. The OCRs were normalized against the total amount of cellular protein. Values shown are means ± S.D. ***P < 0.001 as compared with WT + EV cells, and ^†††^*P* < 0.001 as compared with KO + EV cells (one-way ANOVA with Tukey’s post hoc test).

Next, the mitochondrial oxygen consumption rate (OCR) of C2C12 cells in proliferation condition was analyzed using an extracellular flux analyzer (Fig. 6a). KO + EV cells showed significantly impaired basal respiration, proton leak, ATP-linked respiration, maximal respiration, and reserve capacity (Fig. 6b) as compared to WT + EV cells. These reductions in OCR in KO + EV cells were reversed in KO + StarD7 cells, suggesting that StarD7 is required for the proper maintenance of mitochondrial phospholipid content and respiration activity in C2C12 cells.

### StarD7 is important for myogenic differentiation in human skeletal myoblasts

**Figure 7:**
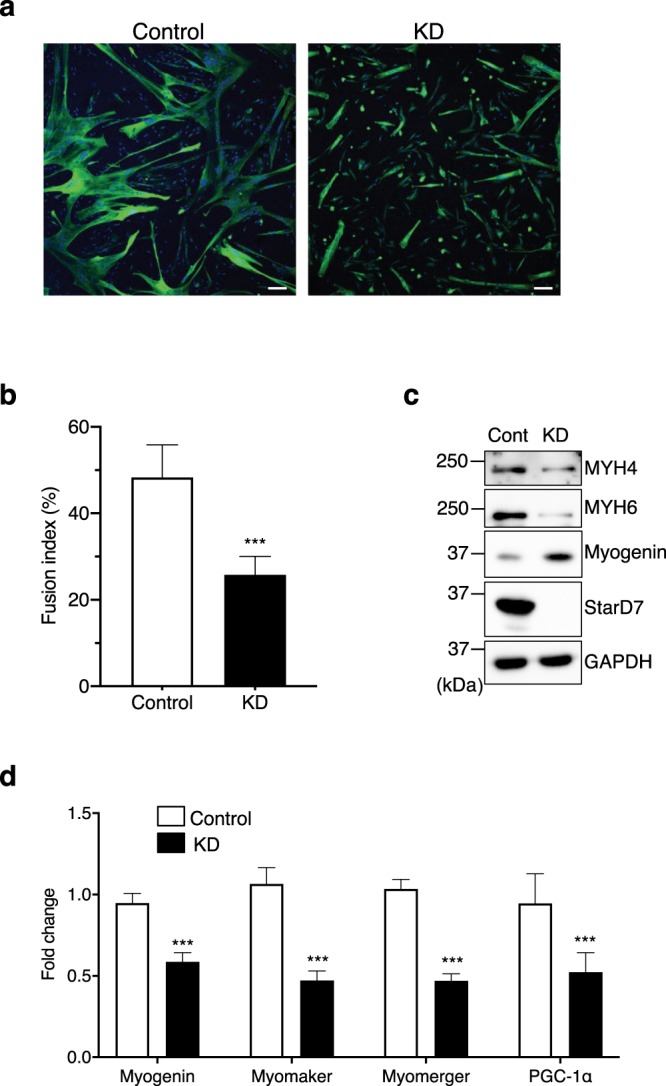
Knockdown of StarD7 impaired myogenic differentiation in human primary myoblasts. (**a**) After transfection with siRNAs against StarD7 (KD) or control siRNA, human primary myoblasts were cultured in differentiation medium for 3 days, then immunostained with anti-MYH6 antibody (green). Nuclei were stained with DAPI (blue). *Bars* indicate 100 μm. (**b**) The fusion indexes were calculated, and are presented as the means ± S.D. ***P < 0.001 as compared with control siRNA (Student’s t test). (**c**) After inducing differentiation, protein levels of MYH4, MYH6, myogenin and StarD7 were analyzed by western blotting. GAPDH was used as a protein loading control. (**d**) The mRNA levels of myogenin, myomaker, myomerger and PGC-1α were quantified by qPCR after inducing differentiation. Data were normalized to the GAPDH level. Values shown are means ± S.D. from three independent culture dishes. ***P < 0.001 as compared with control siRNA (Student’s t test).

Finally, we used primary human skeletal myoblasts to verify that StarD7 is important for myogenic differentiation in human cell lines. After transfection with control or StarD7-targeting siRNA, the cells were cultured in differentiation medium for 3 days and MYH6 was visualized by immunofluorescence. As shown in Fig. 7a, MYH6-positive myotubes were significantly reduced in KD cells. The fusion index of control and KD cells was 48.3% and 25.8%, respectively (Fig. 7b). As shown in Fig. 7c, the reduced expression of MYH4 and MYH6 was also confirmed by western blotting. In contrast to the results for C2C12 cells, the protein content of myogenin was increased in StarD7-silenced human primary myoblasts. Then, we quantified the mRNA level of myogenin, and found that it was significantly reduced in the KD cells (Fig. 7d). These results suggest that the protein level of myogenin could be translationally or post-translationally regulated in human skeletal myoblasts. The mRNA levels of myomaker, myomerger and PGC-1α were significantly decreased in the KD cells (Fig. 7d). Similar results were obtained when another StarD7-targeting siRNA (KD2) was used (Fig. S6). These results suggest that StarD7 is important for myogenic differentiation in primary human skeletal myoblasts as well as C2C12 cells.

## Discussion

Several investigations on the function of StarD7 have used various cell types, such as HEPA-1^14–16^ and HepG2 (hepatocyte)^19^, JEG-3 (trophoblast)^20,21^, BEAS-2B (bronchial epithelium)^18^, HeLa (uterine), and HEK293 (kidney) cells^17^. However, there have been no reports on the biological role of StarD7 on skeletal muscle cells. Therefore, here we studied mouse C2C12 myoblasts and human primary myoblasts and found that StarD7 deficiency caused mitochondrial dysfunction and impaired myotube formation, suggesting that StarD7 is important for muscle cell differentiation.

Energy production by myoblasts is mainly dependent on glycolysis whereas myotubes mainly depend on oxidative phosphorylation to satisfy their high energy requirements. Complete myogenic differentiation therefore requires changing energy generation from cytosolic glycolysis to mitochondrial oxidative phosphorylation^28,29^. Previous studies have demonstrated incomplete myogenesis if mitochondrial dysfunction occurs. For example, mitochondrial inhibitors such as chloramphenicol (an inhibitor of mitochondrial translation)^30^, carbonyl cyanide *p*-(trifluoromethoxy) phenylhydrazone (an uncoupler of mitochondrial oxidative phosphorylation)^31^, rotenone (a mitochondrial complex I inhibitor)^32^, myxothiazol (a mitochondrial complex III inhibitor)^32^ and oligomycin (a mitochondrial ATP synthase inhibitor)^32,33^ inhibit myogenic differentiation in C2C12 cells, leading to the proposal that mitochondria potentially function as a regulator of myogenesis^34^. Taking these findings into consideration, the impairment of myogenic differentiation by the loss of StarD7 is reasonable because this loss attenuates mitochondrial function (Fig. 6).

PC is the most abundant phospholipid in mitochondria is not synthesized within the organelle. Therefore, *de novo* synthesized PC at the ER must be supplied to mitochondria through ER-mitochondria contact sites to maintain mitochondrial PC levels. In this study, we demonstrated that the loss of StarD7 causes a reduction in mitochondrial PC levels (Fig. 5), suggesting that the protein is essential for mitochondrial PC homeostasis. In skeletal muscle, *de novo* PC synthesis is primarily dependent on the CDP-choline pathway (or Kennedy pathway), which is initiated by choline phosphorylation with choline kinase β. It was demonstrated that genetic mutations in the choline kinase β gene cause rostrocaudal muscular dystrophy in mouse^35,36^. In human, mutations in choline kinase β have been found in patients suffering from rare congenital muscular dystrophy^37,38^. Interestingly, skeletal muscle mitochondria in both the mice and patients had reduced levels of PC, morphological abnormalities, and decreased respiratory chain enzymatic activity^37,39^. Taken together, these findings suggest that the maintenance of proper PC levels in mitochondria is important for not only mitochondrial function but also for skeletal muscle differentiation and integrity.

Recent studies have revealed that two muscle-specific membrane proteins, myomaker and myomerger (also referred to as minion or myomixer), play an essential role in myoblast fusion^40–43^. The expression of both transcripts was significantly reduced in StarD7-deficient C2C12 cells and human myoblasts (Figs. 3c, 4e, 7d and S6d), thus possibly explaining impaired myogenic differentiation. In addition to these membrane proteins, we found that the expression of PGC-1α was significantly reduced in StarD7-deficient myoblasts. PGC-1α is a possible master regulator of mitochondrial biogenesis^44^. A previous study using C2C12 cells demonstrated that the lack of PGC-1α results in decreased mitochondrial mass and myogenin expression, an increase in mitophagy, and impaired myogenic differentiation^45^. Thus, the decrease in PGC-1α in *StarD7*-KO cells may contribute to incomplete differentiation. Myogenin is a major muscle-specific transcription factor that promotes muscle differentiation and controls the final differentiation step of myogenesis. Myogenin and MyoD can regulate the transcription of myomaker by binding to its transcription start site in both mouse and chicken^40,46^. Consistent with these reports, we found that the expression of both myogenin and myomaker was significantly reduced in C2C12 cells (Figs. 3b,c, and 4d,e).

In contrast to the results of C2C12 cells, the protein level of myogenin was unexpectedly elevated while the mRNA level of myogenin was reduced in StarD7-silenced human myoblasts (Figs. 7c,d, and S6c,d). The precise mechanism of this inverse correlation between C2C12 cells and human myoblasts is still unknown. Furthermore, myogenin protein levels were decreased more drastically than myogenin mRNA levels in the StarD7-deficient C2C12 cells (Figs. 3b,c, and 4d,e). Possible explanation for this discrepancy is the results of translational or post-translational regulation of myogenin. It was reported that myogenin is an extremely unstable protein (a half-life is approximately 20 min)^47^ and is degraded by proteasome dependent manner after polyubiquitination. Several proteins preventing myogenin degradation from proteasomal degradation have been identified. For example, egl-9, one of the family of hypoxia inducible factor 3 (EGLN3, also known as PHD3, HPH1, and SM 20) was reported to bind to myogenin, and prevent its degradation by a ubiquitin ligase complex containing von Hippel-Lindau (VHL) protein^48^. Protein 4.1 R was also reported to stabilizes myogenin by associating with VHL^49^. Furthermore, TATA-binding protein (TBP)-interacting Protein 120B (TIP120B) inhibits Skp1/Cullin 1/F-box protein (SCF)-dependent ubiquitination of myogenin, and leads to stabilization of the protein^50^. We speculate that these and/or other mechanisms might be involved in the regulation of myogenin protein. Further study might be necessary for understanding the regulation of myogenin protein expression.

Saita *et al*. recently demonstrated that the maturation of StarD7 protein is mediated by the mitochondrial protease PARL in human cell lines such as HEK293 and HeLa^17^. In this study, we established *PARL*-KO HEK293 cells and observed the partial contribution of PARL to StarD7 maturation. However, as shown in this study, loss of PARL had no effect on the maturation of StarD7 in mouse cell lines such as C2C12 and HEPA-1 (Figs. 1e and S4a). These results could suggest differences in the processing and maturation mechanisms of the protein between these human and murine cell types. Additionally, in humans, mitochondrial StarD7 has been reported to be localized in the IMS. In contrast, we demonstrated that the protein localized not only in the inner mitochondrial area but also in the outer leaflet of the OMM using immunofluorescence (Figs. 1c and S2) and immuno-electron microscopy (Figs. 1d and S3). These results support our previous conclusion that StarD7 transfers PC between the ER and mitochondria at organelle contact sites in C2C12 and HEPA-1 cells. Further study is required to identify the mitochondrial proteinases important for StarD7 cleavage in these cells and possibly explain the differences in mitochondrial localization.

Mitochondrial myopathy is characterized by symptoms such as muscle weakness and loss of cardiac function, and is known to be a mitochondrial disease caused by mutations in genes responsible for mitochondrial function and homeostasis. There is no curative therapy for the disease today, and only symptomatic treatment is available. In this study, we demonstrated that StarD7 is important for myotube formation in primary human myoblasts. Mitochondrial myopathy due to StarD7 mutation in human has not been reported to date. Although the number of identified mutations involved in myopathy is increasing, many genes responsible for human myopathy remain unknown. This study will contribute to further understanding skeletal myogenesis and help in the diagnosis and treatment of mitochondrial myopathy.

## Methods

### Antibodies

The antibodies used in this study were: anti-myc antibody (M192-3) from MBL (Nagoya, Japan), anti-SDHA (D6J9M) and anti-tubulin β (9F3#5346) antibodies from Cell Signaling Technology (Danvers, MA), anti-YME1L1 (11510-1-AP) and anti-MYH4 (20140-1-AP) antibodies from Proteintech (Chicago, IL), anti-TOM70 antibody (sc-390545) from Santa Cruz Biotechnology, Inc. (Dallas, TX), Membrane Integrity WB Antibody Cocktail (anti-CVa, -Core1, -porin, -CypD, and -CytC antibodies) and anti-PARL antibody (ab45231) from Abcam (Cambridge, UK), anti-MYH6 (NB300-284) and anti-myogenin (NBP2-32986) antibodies from Norus Biologicals (Littleton, CO), anti-actin from Sigma-Aldrich (St. Louis, MO), anti-V5 antibody from Thermo Fisher Scientific (Waltham, MA), and anti-glyceraldehyde 3-phosphate dehydrogenase (GAPDH) antibody (5A12) from Wako Pure Chemicals (Osaka, Japan). Anti-MyoD antibody (Ab-200) was from GenScript (Piscataway, NJ). Anti-StarD7 antibody was prepared as reported previously^14^.

### Cell culture, differentiation, and siRNA-mediated KD

C2C12 cells and human skeletal myoblasts (Thermo Fisher Scientific) were cultured in DMEM (high glucose) containing 10% FBS at 37 °C in a humidified incubator containing 5% CO_2_. For differentiation into myotubes, the culture medium was replaced with a differentiation medium (DMEM supplemented with 2% horse serum) and the medium was changed every 2 days. Stealth siRNAs (Thermo Fisher Scientific) were used for siRNA-mediated KD of StarD7: (KD1: GCCCUGCUCGGAUUGAGUAUGCUUA and KD2: CAAGAACAUGGAGAUCAAAGUGAAA) for C2C12 cells and (KD: CCUUAUCCAAUGUACUCACGGGAUU and KD2: GGUUCCGAGGUUCUUCACUGGGUAA) for human skeletal myoblasts. Stealth siRNA negative control (Med GC) was used for control experiments. Cells were transfected with these siRNAs using Lipofectamine RNAiMAX (Thermo Fisher Scientific) one day before differentiation, according to the manufacturer’s instructions.

### Immunofluorescence and fusion index

Cells were grown on glass coverslips coated with 1% gelatin (Sigma-Aldrich). An expression vector for StarD7 fused with a myc-tag at the C-terminus was prepared as reported previously^15^. Cells were transfected with the expression vector using Lipofectamine 2000 (Thermo Fisher Scientific) according to the manufacturer’s instructions. The cells were fixed with 4% paraformaldehyde in PBS for 15 min, washed with PBS, permeabilized with 0.005% digitonin (w/v) (Sigma-Aldrich) or 0.1% Triton X-100 (w/v) for 10 min, then blocked with 5% skim milk for 30 min. The cells were treated with primary antibodies overnight at 4 °C, followed by Alexa 488-conjugated labeled secondary antibodies (Thermo Fisher Scientific) for 1 hour at room temperature. Mitochondria were stained with MitoTracker Red CMXRos (Thermo Fisher Scientific) before fixation. Nuclei were stained with 4′,6-diamidino-2-phenylindole (DAPI). Samples were observed with a confocal microscope (LSM780; Zeiss, Oberkochen, Germany). The fusion index was calculated as the percentage of the number of nuclei in MYH6-positive C2C12 cells with 3 or more nuclei divided by the total number of nuclei counted^42^. Five fields were chosen randomly to measure the index.

### Immuno-electron microscopy

C2C12 and HEPA-1 cells were transfected with the expression vector containing StarD7 fused with a V5-tag at the C-terminus using Lipofectamine 3000 (Thermo Fisher Scientific) according to the manufacturer’s instructions. After 24 hours, the cells were fixed with 4% paraformaldehyde containing 0.05% glutaraldehyde (w/v) in PBS for 1 hour. The cells were then dehydrated in increasing concentrations of methanol, embedded in LR Gold resin (Electron Microscopy Sciences, Hatfield, PA) and polymerized under a UV lamp at –20 °C for 24 hours. Ultrathin sections containing cell pellets (80 nm thick) were cut using an ultramicrotome (Ultracut UCT; Leica, Wetzlar, Germany) and collected on nickel grids coated with a collodion film, washed with PBS several times, then incubated in blocking solution (1% normal goat serum, 2% bovine serum albumin, and 50 mM Tris-HCl saline; pH 8.2) for 30 min to block non-specific binding. Sections were then incubated in blocking solution containing a 1:10 dilution of a mouse monoclonal antibody against V5-tag for 1 hour at 37 °C (C2C12 cells) or a 1:100 dilution of the same antibody for 1 hour at room temperature (HEPA-1 cells). After incubation with the primary antibody, the sections were washed with PBS and then incubated with a 1:50 dilution of a goat antibody against mouse IgG conjugated with 10 nm gold particles (BBI Solutions, Cardiff, UK) for 1 hour at room temperature. The sections were rinsed with PBS and deionized-distilled water several times, contrasted with uranyl acetate, and viewed using an H-7650 electron microscope (Hitachi, Tokyo, Japan) operated at 80 kV.

### Isolation of mitochondria and alkaline carbonate extraction

Mitochondria and cytosolic fractions were freshly prepared from cells plated at 70–80% confluence using a Mitochondria Isolation Kit for Cultured Cells (Thermo Fisher Scientific) according to the manufacturer’s instructions. Alkaline carbonate extraction was performed as described previously^15^. Mitochondria were suspended in 0.1 M sodium carbonate buffer, pH 11.5, and incubating on ice for 30 min. Membrane pellets (P) and supernatants (S) were separated by centrifugation at 100,000 × *g* for 30 min at 4 °C. Components in the supernatant were precipitated by adding 10% trichloroacetic acid. The membrane and soluble fractions were dissolved in the same volume of sample buffer (65 μl), then the same volume of each protein solution (15 μl) was separated by SDS-PAGE and analyzed by western blotting.

### Western blotting analysis

Western blotting was performed as described previously^15^. The proteins separated with SDS-PAGE were transferred to PVDF membranes (FluoroTrans, Pall Corp., Port Washington, NY) using a Trans-Blot SD Semi-Dry Transfer blotter (Bio-Rad Laboratories, Hercules, CA), then incubating the membranes with 5% (w/v) skim milk in TBS for 1 hour and washing three times with T-TBS (TBS containing 0.02% Tween 20). The membranes were then incubated with primary antibodies overnight at 4 °C, washed three times with T-TBS, then incubated with horseradish peroxidase–conjugated IgGs for 1 hour at room temperature. The membranes were washed three times with T-TBS and stained with Clarity Western ECL Substrate (Bio-Rad) according to the manufacturer’s instructions and visualized using a ChemiDoc Touch (Bio-Rad).

### Genome editing and rescue experiments

The *StarD7*-KO C2C12 cells were established by genome editing using a vector-based CRISPR/Cas9 system. To reduce the incidence of off-target ablation, we used a Cas9 double-nickase (Cas9n) method^51^. The preparation of sgRNA-Cas9n expressing vectors was reported previously^16^. The sgRNA pairs were mStarD7-B1s (5′- CACCGAAACAGAGGCATGGCCGGGG-3′) and mStarD7-B1r (5′-AAACCCCCGGCCATGCCTCTGTTTC-3′), and mStarD7-B2s (5′-CACCGCGCTCTCCGGTGTTTTCGTA-3′) and mStarD7-B2r (5′-AAACTACGAAAACACCGGAGAGCGC-3′). After co-transfection with an empty vector or a pair of StarD7-targetting vectors using Lipofectamine 2000 transfection reagent, the cells were cultured in the presence of 1.5 µg/ml puromycin for 5 days. Surviving cells were then seeded as single colonies in 96-well plates. Each clone was expanded and screened for StarD7 expression by western blotting, as described above. Genomic mutation was confirmed as described previously. For rescue experiments, *StarD7*-KO C2C12 cells were transfected with an empty or human StarD7-encoding plasmid vector (pCAGGS-hStarD7-I)^14^ using Lipofectamine 2000 and cultured for 1 week with 500 μg/ml G418. Surviving cells were plated into single colonies, and clones expressing the StarD7 protein were selected. We used clone #1 to obtain the data shown in Figs. 3–6 and clone #2 to obtain the data shown in supplemental Fig. S5.

To generate *PARL*-KO mouse cell lines, the sgRNA pairs were cloned into a pSpCas9n(BB)-2A-Puro (PX462) vector (Addgene plasmid 48141) (Addgene, Cambridge, MA). The sgRNA pairs for mouse PARL were mParl-A1-S (5′-CACCGTTGCAAGGTTGGGTACAGCG-3′) and mParl-A1-AS (5′-AAACCGCTGTACCCAACCTTGCAAC-3′), and mParl-A2-S (5′-CACCGTCCCAACCTCTGCCCCACCA-3′) and mParl-A2-AS (5′-AAACTGGTGGGGCAGAGGTTGGGAC-3′). To generate *PARL*-KO HEK293 cells, the sgRNAs hParl-g1S (5′-CACCGAAGTTAGACTTACTTGAGGC-3′) and hParl-g1AS (5′-AAACGCCTCAAGTAAGTCTAACTTC-3′) were cloned into a pSpCas9(BB)-2A-Puro (PX459) V2.0 vector (Addgene plasmid 62988). These vectors were transfected into C2C12, HEPA-1 and HEK293 cells. After puromycin selection, clones lacking PARL protein were screened by western blotting.

### Quantitative real-time PCR (qPCR)

pPCR was performed as described previously^16^. Total RNA was isolated from cells using a RNeasy mini kit (Qiagen), then reverse transcribed using ReverTra Ace qPCR RT Master Mix (TOYOBO, Osaka, Japan) according to the manufacturer’s instructions. qPCR was carried out on a 7300 Real-Time PCR System (Applied Biosystems) using FastStart Universal SYBR Green Master (Roche). GAPDH was used as an internal control for verification. The primers used are listed in Table S1.

### Isolation of mitochondria and quantification of PC by LC-MS/MS

Mitochondria were isolated from cells using a hybrid Percoll-metrizamide gradient method as described previously^16^. Phospholipids were extracted from purified mitochondria (100 µg of protein) according to the Bligh and Dyer method in the presence of 2 µg of internal standards (1,2-dipentadecanoyl PC and 1,2-diheptadecanoyl PE; Avanti Polar Lipids, Alabaster, AL). Lipids were analyzed by reverse-phase ultra-high-pressure liquid chromatography using an Acquity UPLC BEH C18 column (1.7 μm, 2.1 × 50 mm) (Waters, Milford, MA) coupled to a 5500 QTRAP mass spectrometer (Sciex Inc., Framingham, MA) as described in^15^. Phospholipids were detected in multiple reaction monitoring (MRM) mode by selecting the *m/z* of the phospholipid species at Q1 and the precursor ion at Q3. For PC and PE detection, the precursor ion of *m/z* 184 at Q3 in positive ion mode and of *m/z* 196 at Q3 in negative ion mode were monitored, respectively. The content of phospholipid was quantified using MultiQuant, version 2.0 (Sciex), and normalized against the internal standards.

### Measurement of cellular oxygen consumption rate in intact cells

The oxygen consumption rate (OCR) was determined using a Seahorse Extracellular Flux Analyzer XFp (Agilent Technologies, Santa Clara, CA). C2C12 cells (1.2 × 10^4^ cells/well) were plated and cultured in normal medium on a Seahorse plate one day before the assay. On the day of measurement, medium was replaced with 10 mM glucose, 2 mM glutamine, and 1 mM pyruvate–supplemented XF assay medium, and the cell culture plate was placed in a CO_2_-free incubator for 1 h. The OCR was determined using a Seahorse Analyzer in combination with a Cell Mito Stress Test assay kit according to the manufacturer’s instructions. In this assay, 1 μM oligomycin, 0.5 μM carbonyl cyanide 4-(trifluoromethoxy)phenylhydrazone (FCCP), and 0.5 μM rotenone /antimycin A were subsequently added to the assay medium to monitor different aspects of mitochondrial respiration. Cellular protein content was measured using a BCA protein assay kit (Thermo Fisher Scientific), and OCR was normalized for the total amount of cellular protein.

### Statistical analysis

Quantitative data are presented as means ± S.D. Statistical significance was assessed using the Student’s t test or one-way ANOVA with Tukey’s post hoc test. A *P* value of <0.05 was considered statistically significant.

## Supplementary information

Supplementary Information

## References

[1] Vance JE (2015). Phospholipid synthesis and transport in mammalian cells. Traffic.

[2] Lev, S. Nonvesicular lipid transfer from the endoplasmic reticulum. *Cold Spring Harb. Perspect. Biol*. **4**, 10.1101/cshperspect.a013300 (2012). 10.1101/cshperspect.a013300 PMC3475164 23028121

[3] Kornmann B (2009). An ER-mitochondria tethering complex revealed by a synthetic biology screen. Science.

[4] AhYoung AP, Lu B, Cascio D, Egea PF (2017). Crystal structure of Mdm12 and combinatorial reconstitution of Mdm12/Mmm1 ERMES complexes for structural studies. Biochem. Biophys. Res. Commun..

[5] Jeong H, Park J, Jun Y, Lee C (2017). Crystal structures of Mmm1 and Mdm12-Mmm1 reveal mechanistic insight into phospholipid trafficking at ER-mitochondria contact sites. Proc. Natl. Acad. Sci. USA.

[6] Kawano S (2018). Structure-function insights into direct lipid transfer between membranes by Mmm1-Mdm12 of ERMES. J. Cell Biol..

[7] Merkwirth C, Langer T (2008). Mitofusin 2 builds a bridge between ER and mitochondria. Cell.

[8] de Brito OM, Scorrano L (2008). Mitofusin 2 tethers endoplasmic reticulum to mitochondria. Nature.

[9] Szabadkai G (2006). Chaperone-mediated coupling of endoplasmic reticulum and mitochondrial Ca2+ channels. J. Cell Biol..

[10] Iwasawa R, Mahul-Mellier AL, Datler C, Pazarentzos E, Grimm S (2011). Fis1 and Bap31 bridge the mitochondria-ER interface to establish a platform for apoptosis induction. EMBO J..

[11] Hernandez-Alvarez MI (2019). Deficient Endoplasmic Reticulum-Mitochondrial Phosphatidylserine Transfer Causes Liver Disease. Cell.

[12] Clark BJ (2012). The mammalian START domain protein family in lipid transport in health and disease. J. Endocrinol..

[13] Alpy F, Tomasetto C (2014). START ships lipids across interorganelle space. Biochimie.

[14] Horibata Y, Sugimoto H (2010). StarD7 mediates the intracellular trafficking of phosphatidylcholine to mitochondria. J. Biol. Chem..

[15] Horibata Y (2017). Identification of the N-terminal transmembrane domain of StarD7 and its importance for mitochondrial outer membrane localization and phosphatidylcholine transfer. Sci. Rep..

[16] Horibata Y (2016). StarD7 Protein Deficiency Adversely Affects the Phosphatidylcholine Composition, Respiratory Activity, and Cristae Structure of Mitochondria. J. Biol. Chem..

[17] Saita, S. *et al*. PARL partitions the lipid transfer protein STARD7 between the cytosol and mitochondriaz. *EMBO J*. **37**, 10.15252/embj.201797909. Epub 2018 Jan 4 (2018). 10.15252/embj.201797909 PMC5813258 29301859

[18] Yang L (2017). The Phosphatidylcholine Transfer Protein Stard7 is Required for Mitochondrial and Epithelial Cell Homeostasis. Sci. Rep..

[19] Flores-Martin J, Reyna L, Ridano ME, Panzetta-Dutari GM, Genti-Raimondi S (2016). Suppression of StarD7 promotes endoplasmic reticulum stress and induces ROS production. Free Radic. Biol. Med..

[20] Flores-Martin J (2018). Hexosamine pathway regulates StarD7 expression in JEG-3 cells. Mol. Biol. Rep..

[21] Flores-Martin J, Rena V, Marquez S, Panzetta-Dutari GM, Genti-Raimondi S (2012). StarD7 knockdown modulates ABCG2 expression, cell migration, proliferation, and differentiation of human choriocarcinoma JEG-3 cells. PLoS One.

[22] Yang L (2015). Haploinsufficiency for Stard7 is associated with enhanced allergic responses in lung and skin. J. Immunol..

[23] White, C., Nixon, A. & Bradbury, N. A. Determining Membrane Protein Topology Using Fluorescence Protease Protection (FPP). *J. Vis. Exp*. (98), 10.3791/52509 (2015). 10.3791/52509 PMC4541577 25939013

[24] Zhou C (2008). The kinase domain of mitochondrial PINK1 faces the cytoplasm. Proc. Natl. Acad. Sci. USA.

[25] Gonzalez-Baro MR, Granger DA, Coleman RA (2001). Mitochondrial glycerol phosphate acyltransferase contains two transmembrane domains with the active site in the N-terminal domain facing the cytosol. J. Biol. Chem..

[26] Okatsu K, Kimura M, Oka T, Tanaka K, Matsuda N (2015). Unconventional PINK1 localization to the outer membrane of depolarized mitochondria drives Parkin recruitment. J. Cell. Sci..

[27] Liu Y (2017). The Ubiquitination of PINK1 Is Restricted to Its Mature 52-kDa Form. Cell. Rep..

[28] Leary SC, Battersby BJ, Hansford RG, Moyes CD (1998). Interactions between bioenergetics and mitochondrial biogenesis. Biochim. Biophys. Acta.

[29] Remels AH (2010). Regulation of mitochondrial biogenesis during myogenesis. Mol. Cell. Endocrinol..

[30] Seyer P (2011). P43-dependent mitochondrial activity regulates myoblast differentiation and slow myosin isoform expression by control of Calcineurin expression. Exp. Cell Res..

[31] Biswas G (1999). Retrograde Ca2+ signaling in C2C12 skeletal myocytes in response to mitochondrial genetic and metabolic stress: a novel mode of inter-organelle crosstalk. EMBO J..

[32] Pawlikowska P, Gajkowska B, Hocquette JF, Orzechowski A (2006). Not only insulin stimulates mitochondriogenesis in muscle cells, but mitochondria are also essential for insulin-mediated myogenesis. Cell Prolif..

[33] Rochard P (2000). Mitochondrial activity is involved in the regulation of myoblast differentiation through myogenin expression and activity of myogenic factors. J. Biol. Chem..

[34] Wagatsuma A, Sakuma K (2013). Mitochondria as a potential regulator of myogenesis. ScientificWorldJournal.

[35] Sher RB (2006). A rostrocaudal muscular dystrophy caused by a defect in choline kinase beta, the first enzyme in phosphatidylcholine biosynthesis. J. Biol. Chem..

[36] Wu G, Sher RB, Cox GA, Vance DE (2009). Understanding the muscular dystrophy caused by deletion of choline kinase beta in mice. Biochim. Biophys. Acta.

[37] Mitsuhashi S, Nishino I (2011). Phospholipid synthetic defect and mitophagy in muscle disease. Autophagy.

[38] Oliveira J (2015). New splicing mutation in the choline kinase beta (CHKB) gene causing a muscular dystrophy detected by whole-exome sequencing. J. Hum. Genet..

[39] Mitsuhashi S (2011). Muscle choline kinase beta defect causes mitochondrial dysfunction and increased mitophagy. Hum. Mol. Genet..

[40] Millay DP, Sutherland LB, Bassel-Duby R, Olson EN (2014). Myomaker is essential for muscle regeneration. Genes Dev..

[41] Bi P (2017). Control of muscle formation by the fusogenic micropeptide myomixer. Science.

[42] Leikina E (2018). Myomaker and Myomerger Work Independently to Control Distinct Steps of Membrane Remodeling during Myoblast Fusion. Dev. Cell..

[43] Sampath SC, Sampath SC, Millay DP (2018). Myoblast fusion confusion: the resolution begins. Skelet Muscle.

[44] Arany Z (2008). PGC-1 coactivators and skeletal muscle adaptations in health and disease. Curr. Opin. Genet. Dev..

[45] Baldelli S, Aquilano K, Ciriolo MR (2014). PGC-1alpha buffers ROS-mediated removal of mitochondria during myogenesis. Cell. Death Dis..

[46] Luo W, Li E, Nie Q, Zhang X (2015). Myomaker, Regulated by MYOD, MYOG and miR-140-3p, Promotes Chicken Myoblast Fusion. Int. J. Mol. Sci..

[47] Edmondson DG, Brennan TJ, Olson EN (1991). Mitogenic repression of myogenin autoregulation. J. Biol. Chem..

[48] Fu J, Menzies K, Freeman RS, Taubman MB (2007). EGLN3 prolyl hydroxylase regulates skeletal muscle differentiation and myogenin protein stability. J. Biol. Chem..

[49] Huang SC, Zhou A, Nguyen DT, Zhang HS, Benz EJ (2016). Protein 4.1R Influences Myogenin Protein Stability and Skeletal Muscle Differentiation. J. Biol. Chem..

[50] Shiraishi S (2007). TBP-interacting protein 120B (TIP120B)/cullin-associated and neddylation-dissociated 2 (CAND2) inhibits SCF-dependent ubiquitination of myogenin and accelerates myogenic differentiation. J. Biol. Chem..

[51] Ran FA (2013). Double nicking by RNA-guided CRISPR Cas9 for enhanced genome editing specificity. Cell.

