# Supplementary material for: The phosphatidylcholine transfer protein StarD7 is important for myogenic differentiation in mouse myoblast C2C12 cells and human primary skeletal myoblasts
Source: Sci Rep. 2020 Feb 18;10:2845. doi: 10.1038/s41598-020-59444-y (PMC7029042; doi:10.1038/s41598-020-59444-y)
Supplement: Supplementary file 1 — Supplementary Information [file 41598_2020_59444_MOESM1_ESM.pdf]

Supplemental Information

The phosphatidylcholine transfer protein StarD7 is important for myogenic differentiation in mouse myoblast C2C12 cells and human primary skeletal myoblasts

Yasuhiro Horibata, Satomi Mitsuhashi, Hiroaki Shimizu, Sho Maejima, Hirotaka Sakamoto, Chieko Aoyama, Hiromi Ando and Hiroyuki Sugimoto

Table S1: List of primer sequences for qPCR

Target Gene	Sequence
mouse GAPDH	F: TGTGTCCGTCGTGGATCTGA<br>R: TTGCTGTTGAAGTCGAGGAG
human GAPDH	F: AACGGGAAGCTTGTCATCAA<br>R: TGGACTCCACGACGTACTCA
mouse PGC-1α	F: AACCAGTACAACAATGAGCCTG<br>R: AATGAGGGCAATCCGTCTTCA
human PGC-1α	F: GGCAGAAGGCAATTGAAGAG<br>R: TCAAAACGGTCCCTCAGTTC
mouse MYH4	F: GCAGGACTTGGTGGACAAAC<br>R: ACTTGGCCAGGTTGACATTG
mouse MYH6	F: GCTGGAAGATGAGTGCTCAGAG<br>R: CCAGCCATCTCCTCTGTTAGGT
mouse Myogenin	F: CAGTGAATGCAACTCCCACAG<br>R: TGGACGTAAGGGAGTGCAGA
human Myogenin	F: CACTCCCTCACCTCCATCGT<br>R: CATCTGGGAAGGCCACAGA
mouse Myomaker	F: ATCGCTACCAAGAGGCGTT<br>R: CACAGCACAGACAAACCAGG
human Myomaker	F: CCTCATCATCGCGGCAAAGT<br>R: GTGTAGTCCCAGTCCTCAAAGAAG
mouse Myomerger (short form)	F: CAGGAGGGCAAGAAGTTCAG<br>R: ATGTCTTGGGAGCTCAGTCG
mouse Myomerger (long form)	F: ACCAGCTTTCATGCCAGAAG<br>R: ATGTCTTGGGAGCTCAGTCG
human Myomerger	F: ATCCTCATCATCGCGGCAAA<br>R: CCCAGTCCTCAAAGAAGAAGCG

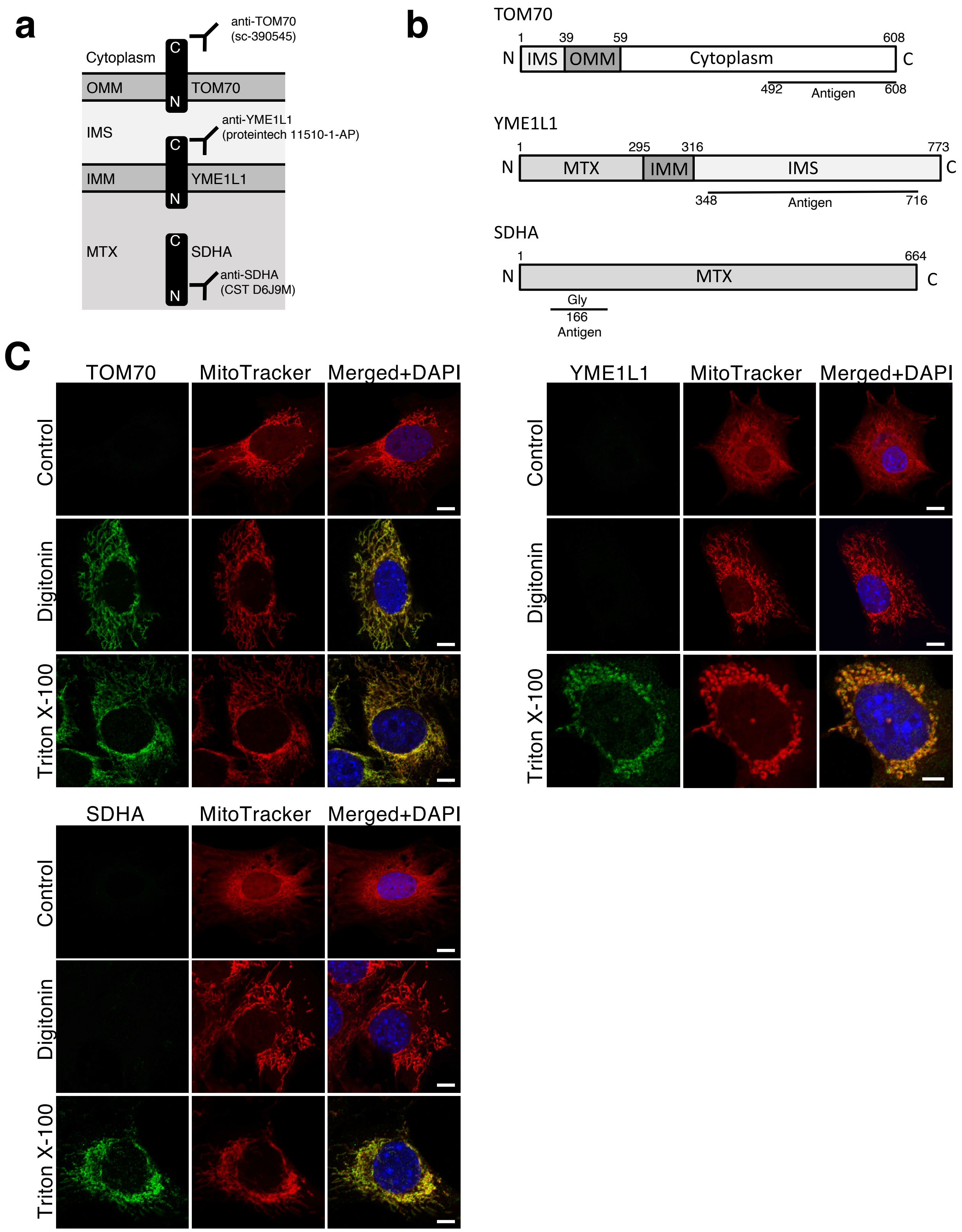

FIGURE. S1. Mitochondrial topologies of TOM70, YME1L1 and SDHA, and assessment of membrane integrity in C2C12 cells.

(a and b) Sub-mitochondrial localization of TOM70, YME1L1 and SDHA. Antibodies and antibody-recognition sites are summarized. Anti-TOM70 antibody (sc-390545) binds to the cytoplasmic tail of TOM70 distributing on the cytoplasmic face of the OMM; anti-YME1L1 antibody (Proteintech 11510-1-AP) binds to the C-terminal region of YME1L1 distributing in intermembrane space (IMS); anti-SDHA antibody (CST D6J9M) binds to the N-terminal region of SDHA distributing inside the matrix (MTX). (c) The integrity of the mitochondrial membrane after permeabilization by 0.005% digitonin (w/v) or 0.1% Triton X-100 (w/v), was assessed by immunostaining in C2C12 cells in proliferation condition. TOM70, YME1L1 and SDHA were immunostained with each antibody (green). Mitochondria and nuclei were stained with MitoTracker Red (red) and DAPI (blue), respectively. Bars indicate 5 µm.

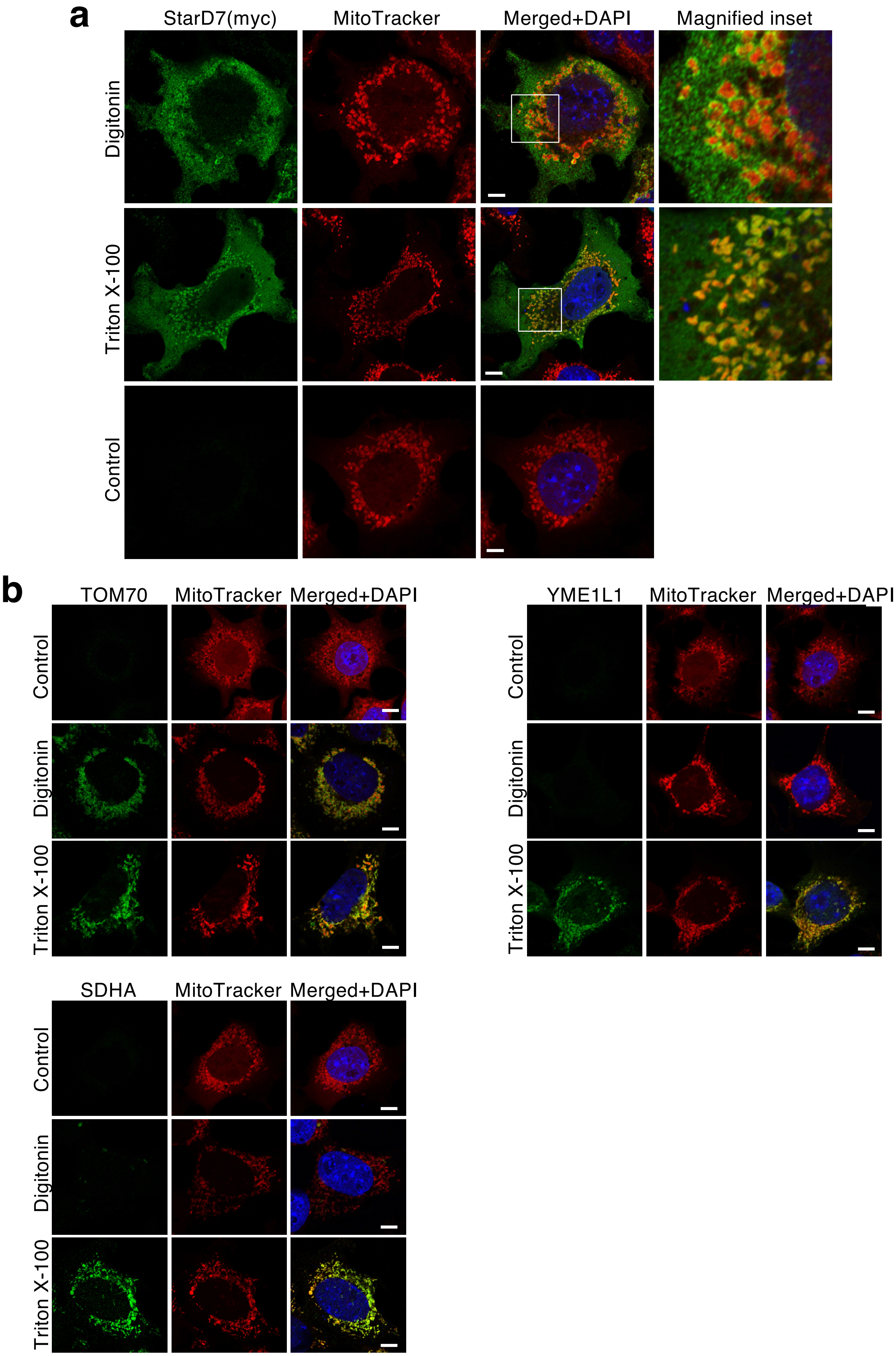

FIGURE. S2. Sub-mitochondrial distribution of StarD7 in HEPA-1 cells.

(a) HEPA-1 cells were transfected with the expression vector for StarD7 fused with a myc tag at the C-terminus. Cells were permeabilized with 0.005% digitonin (w/v) or 0.1% Triton X-100 (w/v), then immunostained with anti-myc antibody (green). Mitochondria and nuclei were stained with MitoTracker Red (red) and DAPI (blue), respectively. (b) Assessment of mitochondrial membrane integrity. Bars indicate 5 μm.

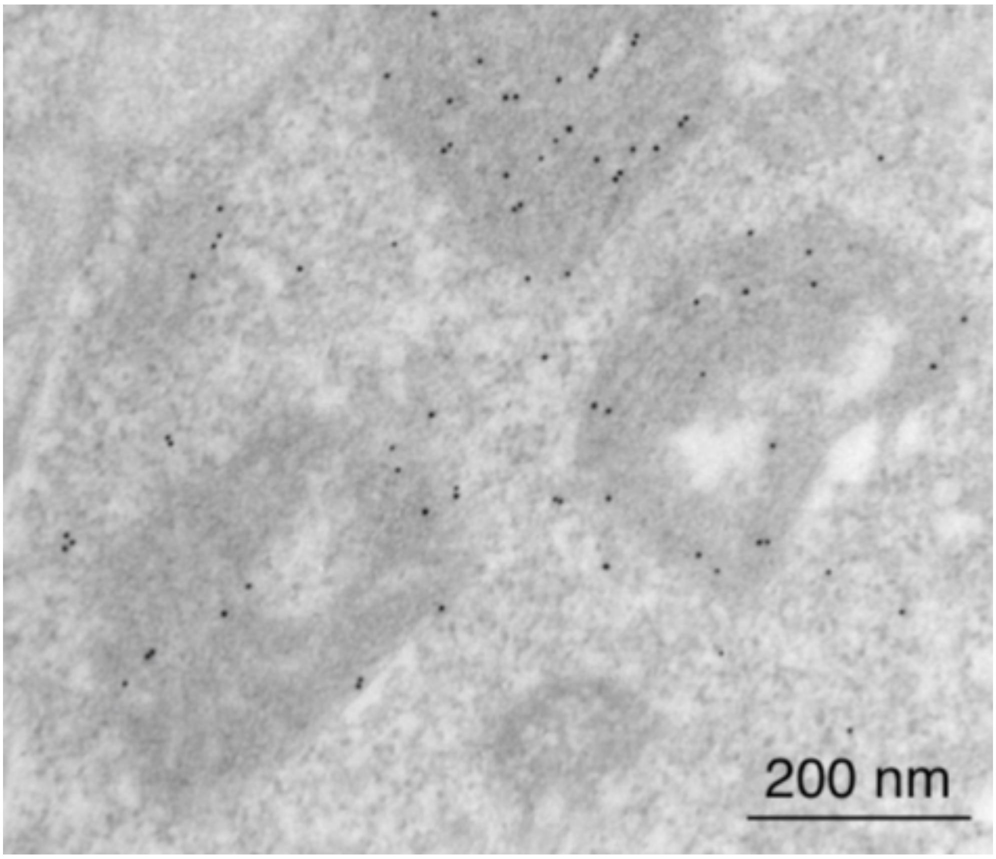

FIGURE. S3. Immuno-electron microscopy of StarD7 in HEPA-1 cells.
After transfection with the expression vector containing StarD7 fused with a V5-tag at the C-terminus, HEPA-1 cells were fixed using paraformaldehyde-glutaraldehyde solution. Sections were incubated with a primary anti-V5 antibody, followed by a secondary gold-conjugated antibody.

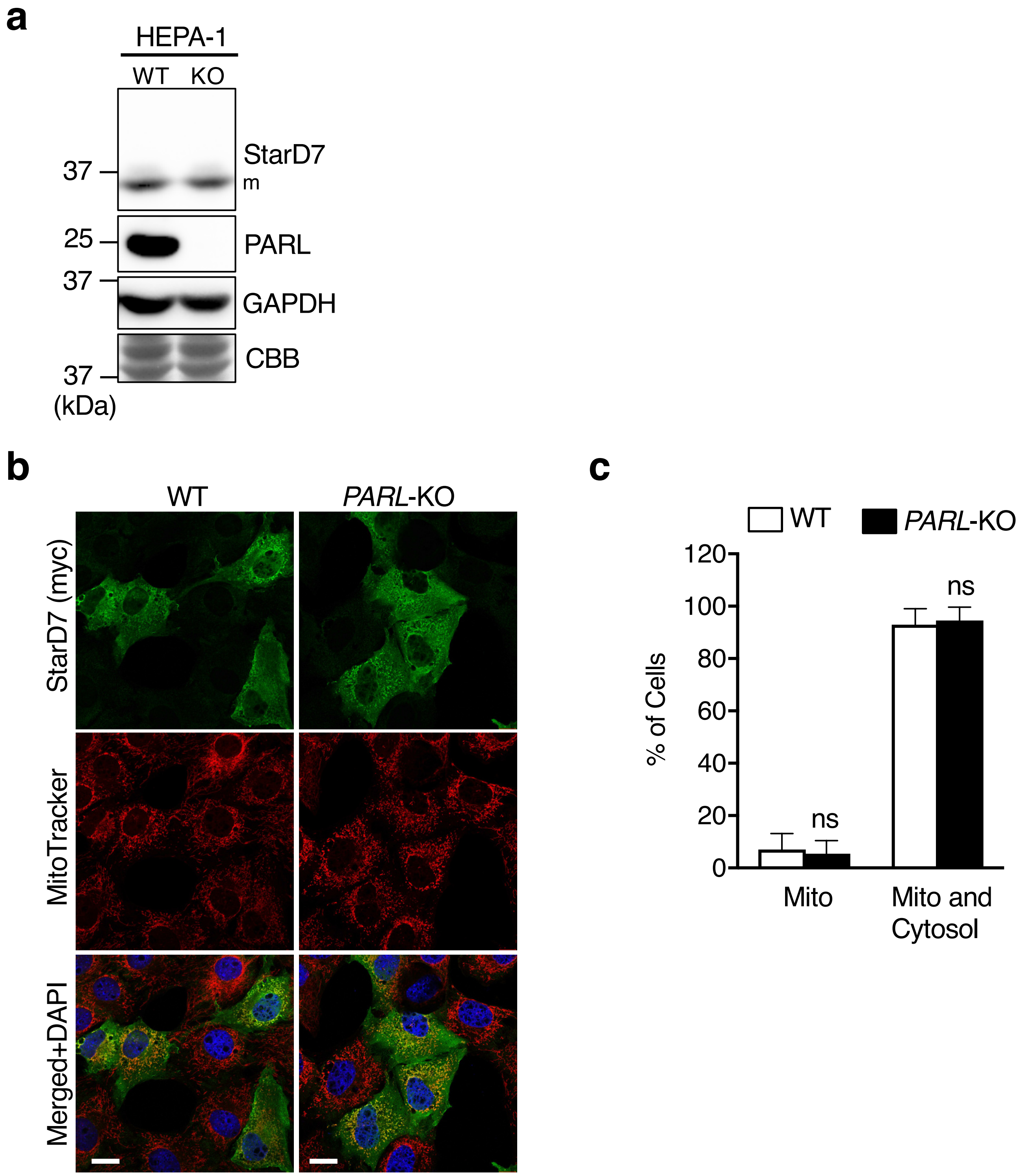

FIGURE. S4. PARL is not involved in the maturation of StarD7 in HEPA-1 cells or in the mitochondria-cytoplasm distribution of the protein in C2C12 cells.

(a) Lysates from WT and *PARL*-KO HEPA1 cells were separated by SDS-PAGE, then the proteins were analyzed by western blotting using anti-StarD7 and PARL antibodies. GAPDH and CBB staining were used as protein loading control. m, mature form of StarD7. (b) WT and *PARL*-KO C2C12 cells were transfected with the expression vector for StarD7 fused with a myc tag at the C-terminus. Cells were permeabilized with 0.1% Triton X-100 (w/v), then immunostained with anti-myc antibody (green). Mitochondria and nuclei were stained with MitoTracker Red (red) and DAPI (blue), respectively. Bars indicate 10 µm. (c) Quantification of b. The percentage of cells showing StarD7 localization only in mitochondria or in both mitochondria and cytosol were calculated. Values shown are means ± S.D. ns indicates not significant as compared to WT (Student's t test).

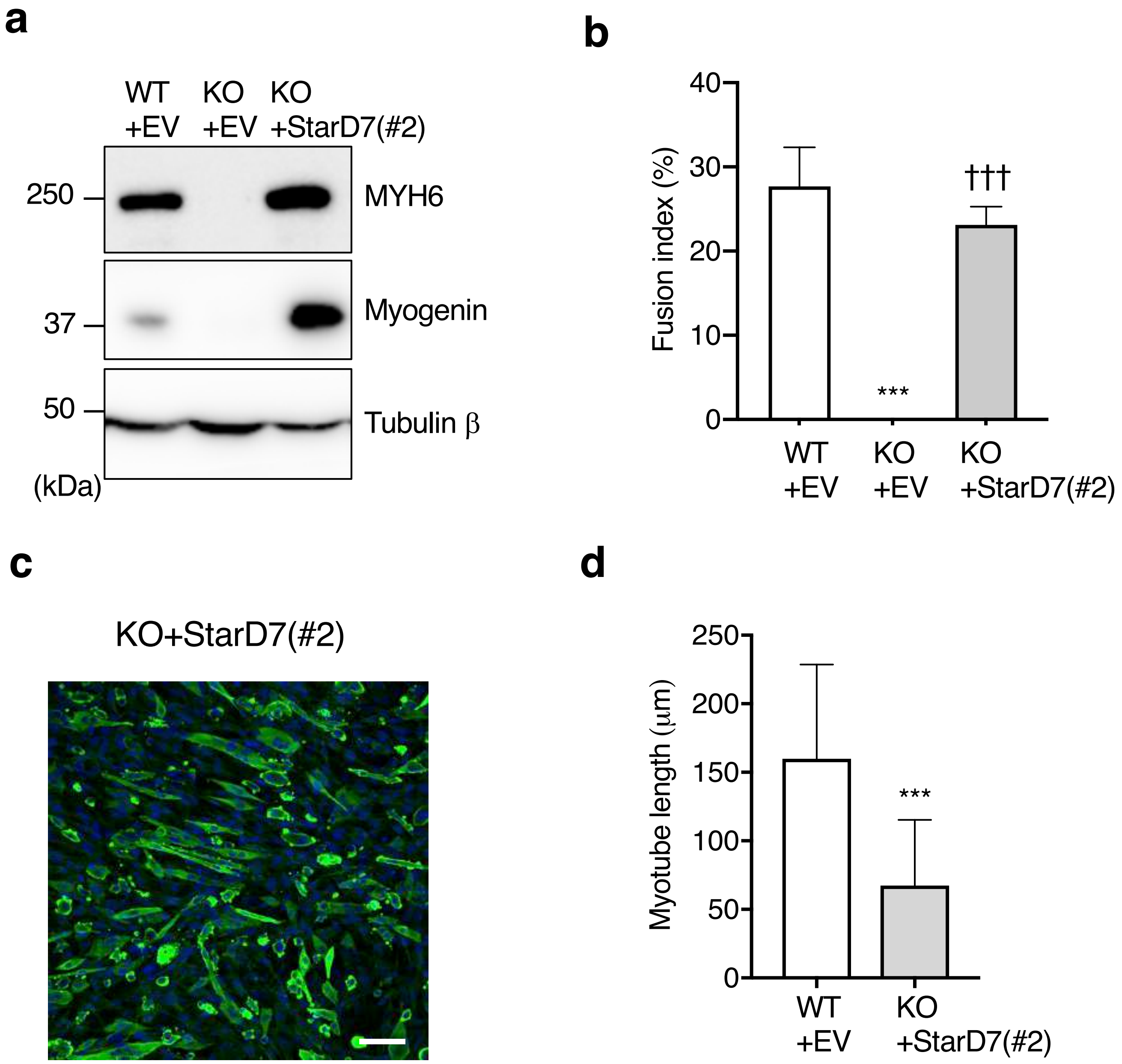

FIGURE. S5. Recovery of myogenic differentiation in KO+StarD7 clone #2.

(a) WT+EV, KO+EV and KO+StarD7 (#2) cells were cultured in differentiation medium for 5 days. Cell lysates were separated by SDS-PAGE and analyzed by western blotting using anti-MYH6 and myogenin antibodies. Tubulin β was used as a protein loading control. (b) The fusion indexes were calculated and are presented as the means ± S.D. *** $P < 0.001$ as compared with WT+EV cells, and ††† $P < 0.001$ as compared with KO+EV cells (one-way ANOVA with Tukey's post hoc test). (c) Cells were immunostained with anti-MYH6 antibody (green). Nuclei were stained with DAPI (blue). Bars indicate 50 μm. (d) Myotube lengths were measured and the results are presented as the means ± S.D. *** $P < 0.001$ as compared with WT+EV cells (Student's t test). Myotube lengths of KO+EV are not shown because the cells were not differentiated.

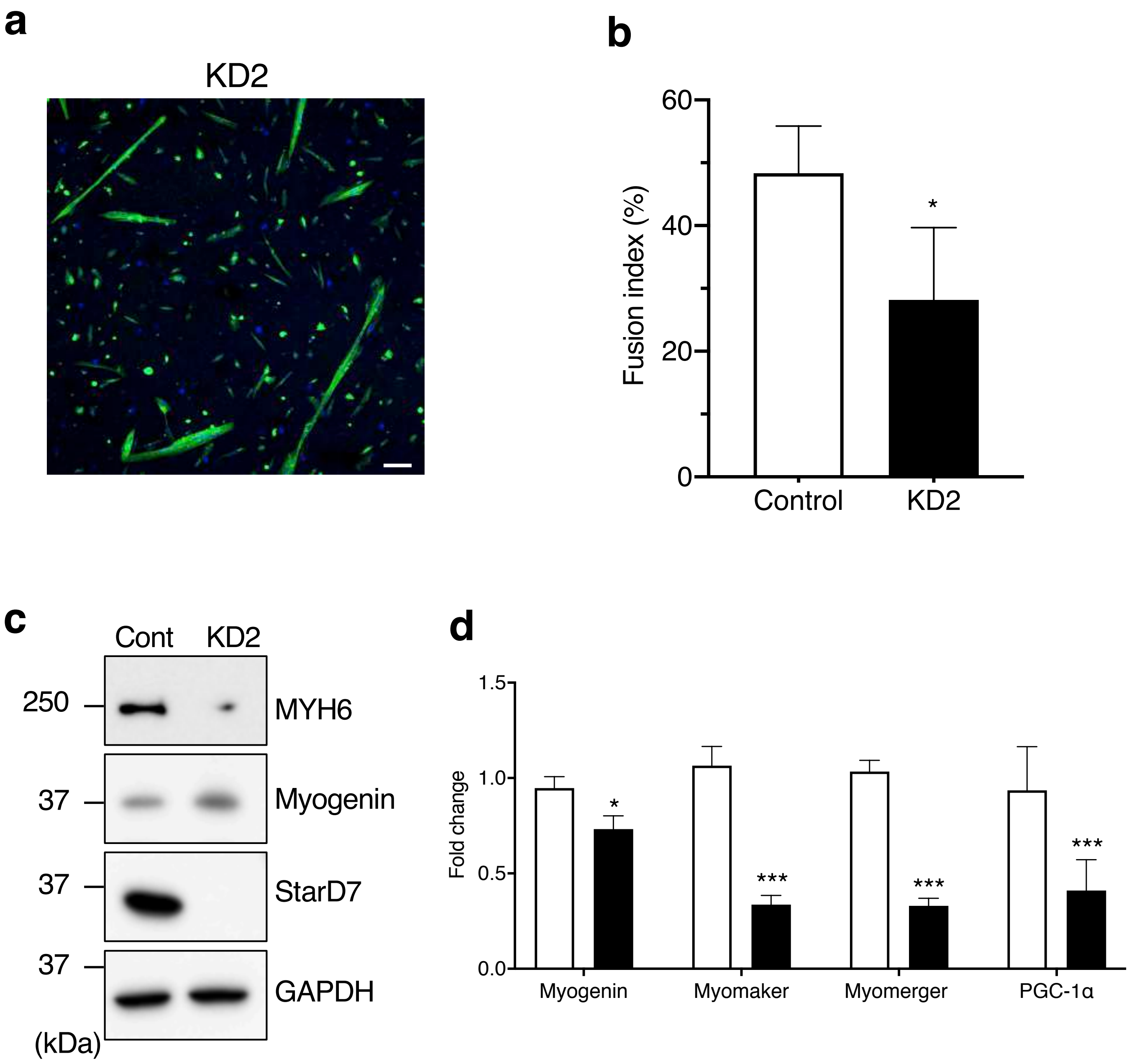

FIGURE. S6. Loss of StarD7 by siRNA (#2) reduced myoblast differentiation in human primary myoblasts.

(a) After transfection with siRNA against StarD7 (KD2) or control siRNA, human primary myoblasts were cultured in differentiation medium for 3 days, then the cells were immunostained with anti-MYH6 antibody (green). Nuclei were stained with DAPI (blue). Bars indicate 100 µm. (b) The fusion indexes were calculated and are presented as the means ± S.D. * $P < 0.05$ as compared with control siRNA (Student's t test). (c) Protein levels of MYH6, myogenin and StarD7 were analyzed by western blotting. GAPDH was used as a protein loading control. (d) The mRNA levels of myogenin, myomaker, myomerger, and PGC-1α were quantified by qPCR. Data were normalized to the GAPDH levels. Values shown are means ± S.D. from three independent culture dishes. * $P < 0.05$ and *** $P < 0.001$ as compared with control siRNA (Student's t test).

Fig.1a

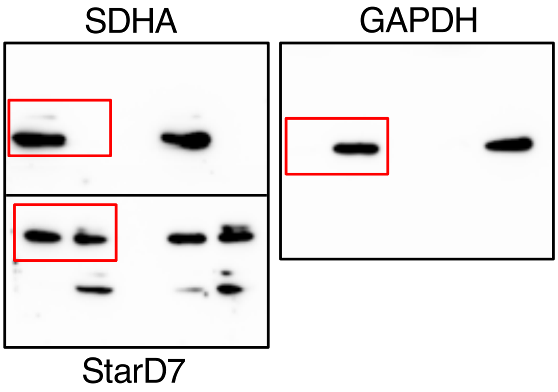

Fig.1b

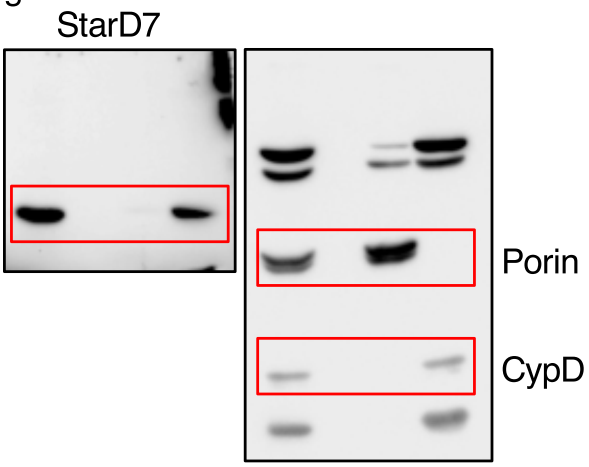

Fig.1e

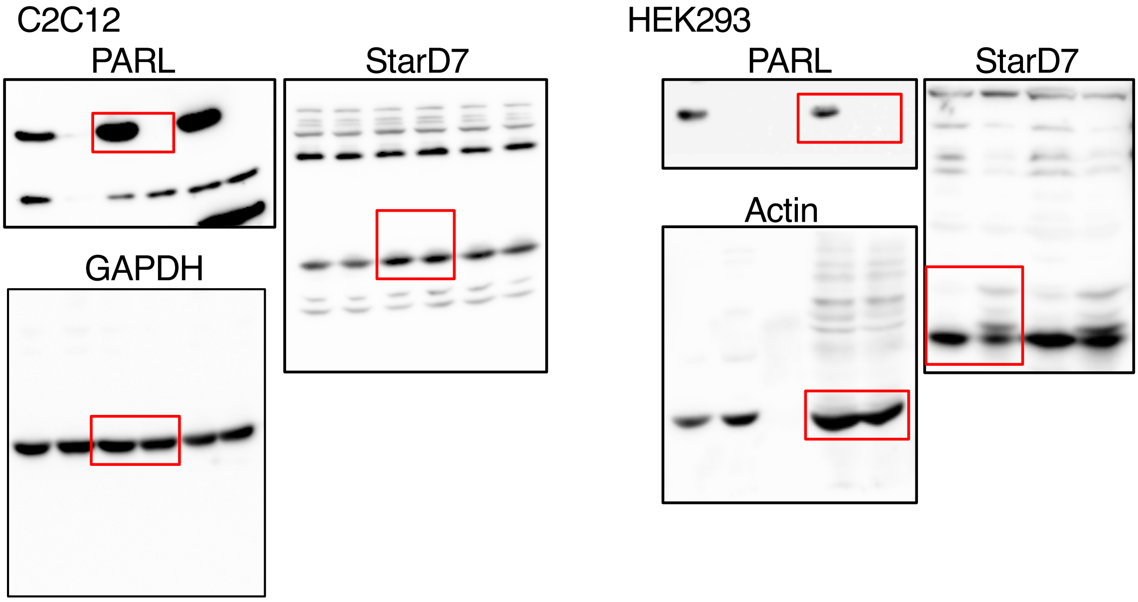

Fig. 2a

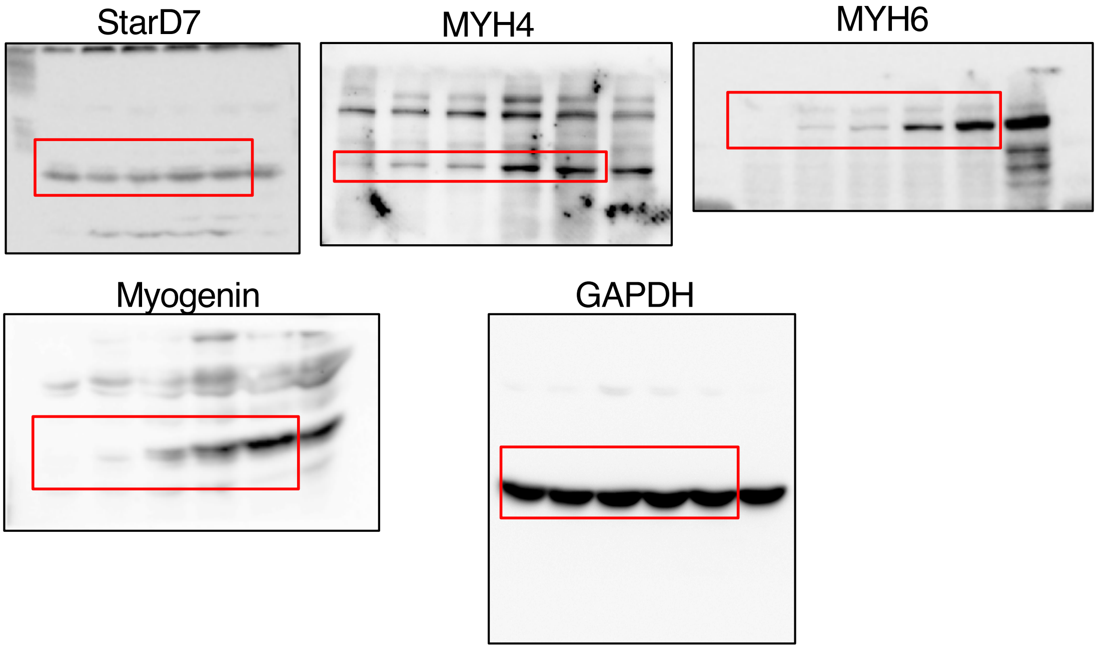

FIGURE. S7. Unprocessed Western blots for the figures and supplemental figures

Fig. 3b

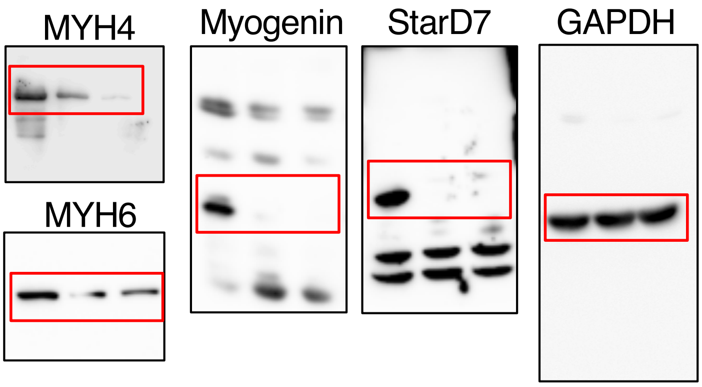

Fig. 3d

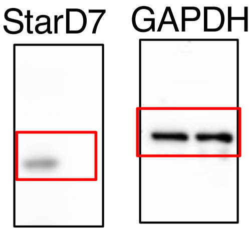

Fig.4a

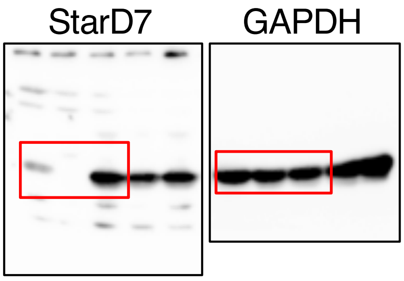

Fig.4d

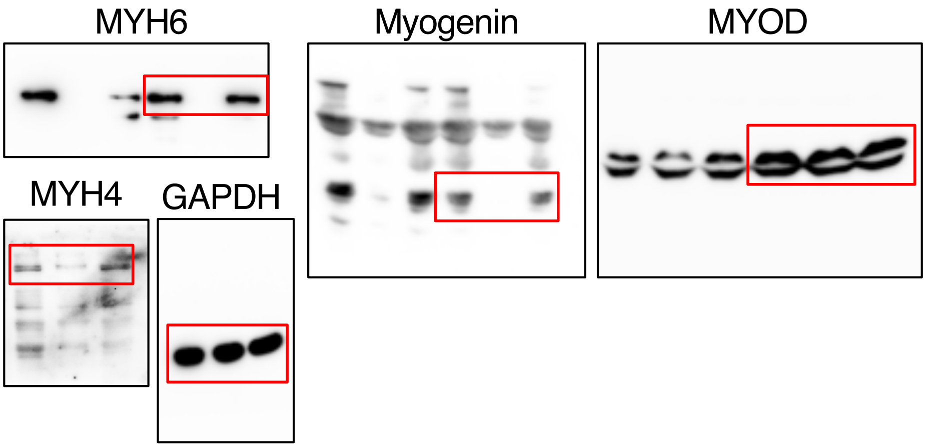

Fig.7c

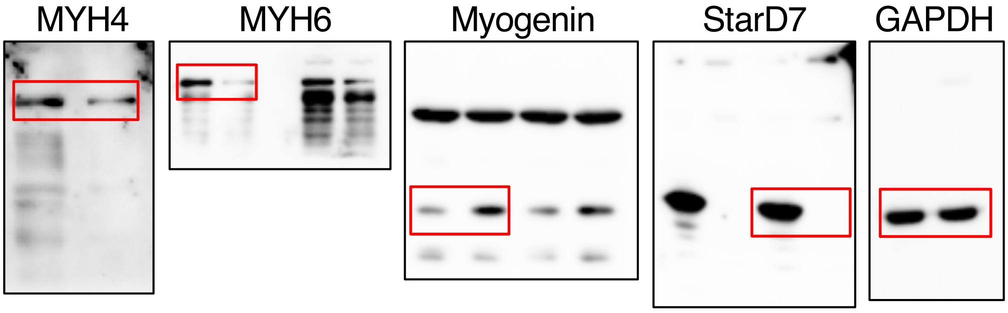

Fig.S4a

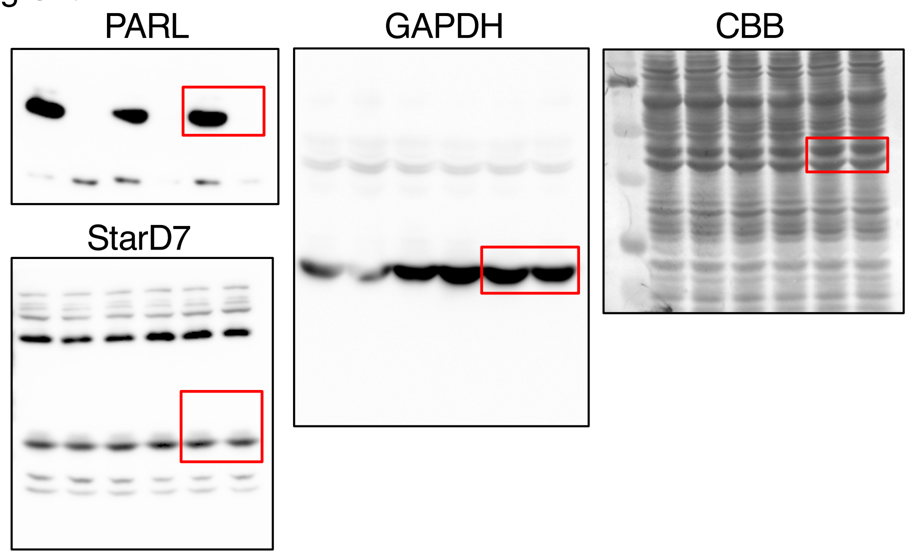

FIGURE. S7. Continued

Fig.S5a

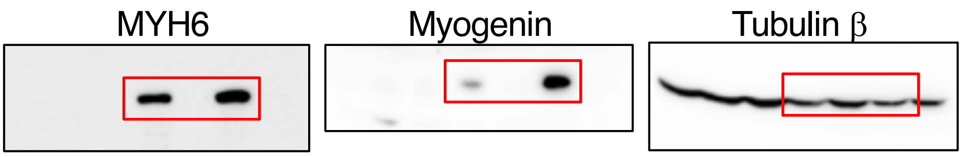

Fig.S6c

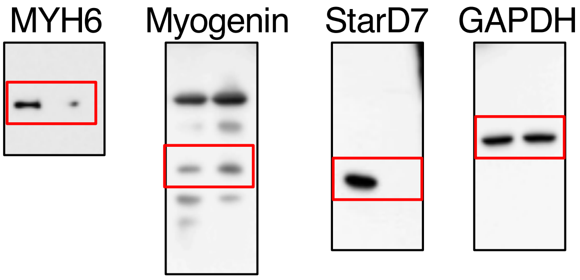

FIGURE. S7. Continued
